# Supplementary material for: Spotting false news and doubting true news: a systematic review and meta-analysis of news judgements
Source: Nat Hum Behav. 2025 Feb 21;9(4):688–99. doi: 10.1038/s41562-024-02086-1 (PMC12018262; doi:10.1038/s41562-024-02086-1)
Supplement: Supplementary file 1 — Sections A–J. [file 41562_2024_2086_MOESM1_ESM.pdf]

nature human behaviour

Supplementary information

https://doi.org/10.1038/s41562-024-02086-1

Spotting false news and doubting true news: a systematic review and meta-analysis of news judgements

In the format provided by the authors and unedited

Contents

Appendix A
: Effect sizes 2
Preregistered analyses . . . 2
Alternative effect sizes . . . 2
Effects on original scales . . . 3

Appendix B
: Moderators 5
Not preregistered moderators . . . 5

Appendix C
: Individual level data 12
How skilled were individual participants? . . . 12

Appendix D
: Binary vs. continuous scales 16
Meta-regression . . . 16
Dichotomizing likert scale responses . . . 16
Binary response scales . . . 18
Odds ratios . . . 19
Meta-analyses . . . 19

Appendix E
: Publication bias 22

Appendix F
: Country comparison 23

Appendix G
: Selection bias 26

Appendix H
: Signal Detection Theory 29

Appendix I
: Detailed search strings 31
First database search . . . 31
Second database search . . . 31

Appendix J
: Included studies 33

Appendix A
Effect sizes

Preregistered analyses

In the main analysis that we report in the paper, we relied on Cohen's d as a standardized effect measure. However, we had pre-registered relying on standardized mean changes using change score standardization (SMCC)[70] for within participant designs, and Hedge's g for effect sizes from between participant designs[73].

As Cohen's d, the SMCC expresses effects in units of (pooled) standard deviations, allowing for comparison across different scales. Also similar to the Cohen's d we calculated, the SMCC relies on a correlation estimate to account for statistical dependencies arising from the within participant design used by most studies. By contrast, the SMCC also uses this correlation coefficient in calculating the pooled standard deviation (and not only the standard error, as with our Cohen's d). As a result, the effect size estimate itself (and not only its certainty) are affected by the imputed correlation value.

Precisely, the SMCC is calculated as

$$SMCC = \frac{MD}{SD_d}$$

with MD being the mean difference/change score (mean true news score minus mean false news score) and SD_d being standard deviation of the difference/change scores, which (assuming equal standard deviations for false and true news) is calculated as: $SD_d = SD_{false/true}\sqrt{2(1-r)}$[74].

The SMCC varies with the imputed correlation value r, because SD_d varies as a function of r. If r is greater than .5, SD_d will be smaller than $SD_{false/true}$, and as a result, the SMCC will be larger than the estimate obtained by Cohen's d. By contrast, when the correlation is less than .5, SD_d will be greater than $SD_{false/true}$, and the SMCC will be smaller than Cohen's d[74]. In our case, the imputed average correlation is 0.26.

Table A1 shows that the SMCC yields slightly smaller effect sizes than the Cohen's d (because the correlation between true and false news is smaller than .5), but all conclusions remain the same.

Alternative effect sizes

Table A1 shows the meta-analytic averages for different effect size estimators for both discernment (H1) and skepticism bias (H2). Besides Cohen's d, the estimator of the main study, and SMCC, the pre-registered estimator, we additionally included the estimates for two alternative estimators: A standardized mean difference assuming independence (SMD), precisely Hedge's g (a version of Cohen's d that corrects for small sample sizes), and a standardized mean change using raw (instead of change) score standardization (SMCR)[75]. When using raw score standardization, the standardized mean change expresses the effect size in terms of the standard deviation units of the pre-treatment (in our case false news) scores, rather than the standard deviation of the difference scores (involving the correlation)[75].

Table A1

Comparison of meta-analytic averages for different effect size estimators.

	Main estimator Cohen's d		*Preregistered estimator* SMCC		*Alternative estimators* SMCR		SMD	
	Discernment	Skepticism bias	Discernment	Skepticism bias	Discernment	Skepticism bias	Discernment	Skepticism bias
Estimate	1.116	0.315	0.917	0.254	1.181	0.328	1.117	0.315
	z = 20.794	z = 8.109	z = 20.893	z = 7.856	z = 20.225	z = 8.178	z = 20.792	z = 8.110
	p = <0.001	p = <0.001	p = <0.001	p = <0.001	p = <0.001	p = <0.001	p = <0.001	p = <0.001
Num.Obs.	302	302	302	302	302	302	302	302
AIC	464.4	504.7	342.8	391.9	505.0	518.3	464.4	504.6
BIC	475.6	515.8	354.0	403.0	516.2	529.4	475.5	515.8

Note: Cohen's d is the estimator we report in the main analysis. SMCC (Standardized mean change using change score standardization) is the estimator we pre-registered. For reference, we provide the results we obtain when using a standardized mean difference assuming independence for all effect sizes (SMD), precisely Hedge's g, and a standardized change score using raw (instead of change) standardization (SMCR). For effects from studies that used a between participant design, we calculated Hedge's g in the results listed under "SMCC" and "SMCR". No adjustments have been made.

Table A2

Frequency table of scales.

	1-point	10-point	100-point	21-point	4-point	5-point	6-point	7-point	binary
Papers	3	2	1	1	21	1	12	12	19
Samples	25	3	1	2	45	19	28	37	37
Effects	25	3	1	2	106	19	41	51	55

Note. A 1-point scale means that values were standardized by the original authors to range from 0 to 1, but have originally been asked on Likert scales.

Among all estimators, the SMCC is the only one in which the effect size estimate depends on the value of the correlation between the false and true news scores. The interpretation of all these standardized effect measures is similar: all are expressed in terms of standard deviations. Yet, they are different estimators, because they rely on different standard deviations, thereby producing different estimates and standard errors[74]. Due to the low average correlation between false and true news ratings, the SMCC produces the smallest effect estimates for both discernment and skepticism bias.

Effects on original scales

Table A3 shows estimates by scale, in the original units of the scale. The table is intended to help interpret the magnitude of the effect sizes reported in the main findings. Note that some scales occur very rarely only (see Tab. A2).

Table A3

(Raw) Mean Differences between true and false news

	4-point	10-point	binary	7-point	6-point	1-point	21-point	5-point
Discernment								
Estimate	0.812	2.440	0.309	1.542	1.100	0.290	3.249	0.700
	z = 15.132	z = 14.304	z = 10.983	z = 9.398	z = 17.178	z = 12.784	z = 7.848	z = 11.737
	p = <0.001	p = <0.001	p = <0.001	p = <0.001	p = <0.001	p = <0.001	p = <0.001	p = <0.001
Num.Obs.	105	2	54	50	40	24	1	18
AIC	26.9	6.2	-73.1	129.8	32.6	-32.8	8.5	7.7
BIC	34.9	2.3	-67.1	135.5	37.7	-29.3	2.5	10.4
Skepticism bias								
Estimate	0.299	-1.807	0.086	-0.025	0.656	0.092	4.361	0.299
	z = 4.883	z = -1.676	z = 3.732	z = -0.238	z = 5.126	z = 5.407	z = 5.083	z = 4.481
	p = <0.001	p = 0.094	p = <0.001	p = 0.812	p = <0.001	p = <0.001	p = <0.001	p = <0.001
Num.Obs.	105	2	54	50	40	24	1	18
AIC	105.4	17.3	-33.2	108.6	104.9	-47.2	11.4	12.0
BIC	113.3	13.3	-27.3	114.3	109.9	-43.7	5.4	14.7

Note: Results of separate meta-analyses for different response scales. The effect sizes are not standardized, so that estimates are to be interpreted on their respective scale. One scale, a 100-point scale, does not appear since there was only one effect size using that scale. A 1-point scale means that values were standardized by the original authors to range from 0 to 1, but have originally been asked on Likert scales. No adjustments have been made.

Appendix B
Moderators

All moderator analyses, with the exception of political concordance, only reveal statistical associations, not causal effects, because the moderator variables vary mostly between studies: For example, some studies provided news sources, while others did not. But these studies differ in many other ways, all of which potentially confound any observed association.

Table B1 shows the results of the different meta regressions by moderator variable on discernment and Table B2 on skepticism bias. Figures B1 and B2 visualize those results by showing the distribution of effect sizes by moderator variable.

Not preregistered moderators.

Scale symmetry. First, to avoid biasing our estimate for H2, we removed one study[62] that used a very asymmetrical set of answer options asked participants ("According to your knowledge, how do you rate the following headline? 1—not credible; 2—somehow credible; 3—quite credible; 4—credible; 5—very credible"). Second, we coded whether the remaining scales were perfectly symmetrical or not. Table B3 shows the frequency by which both scale types occurred.

Perfectly symmetrical scales include all binary scales (e.g. "True" or "False", "Real" or

Table B1
Moderator effects on Discernment

	Country	Concordance	Family	Format	Source	Scale	Symmetrie	False news	All
intercept	0.992<br>z = 12.825<br>p = <0.001	0.594<br>z = 7.372<br>p = <0.001	1.256<br>z = 11.759<br>p = <0.001	1.086<br>z = 13.478<br>p = <0.001	1.280<br>z = 13.445<br>p = <0.001	1.282<br>z = 11.963<br>p = <0.001	1.391<br>z = 13.156<br>p = <0.001	1.124<br>z = 20.286<br>p = <0.001	0.986<br>z = 6.173<br>p = <0.001
Country: US (vs. nonUS)	0.228<br>z = 2.142<br>p = 0.033								-0.098<br>z = -1.004<br>p = 0.330
Political Concordance : Discordant (vs. Concordant)		0.078<br>z = 1.717<br>p = 0.097							0.070<br>z = 1.129<br>p = 0.276
News family: Other (vs. Covid)			-0.005<br>z = -0.030<br>p = 0.976						
News family: Political (vs. Covid)			-0.257<br>z = -1.984<br>p = 0.049						
News Format: Headline & Picture (vs. Headline)				-0.005<br>z = -0.039<br>p = 0.969					-0.506<br>z = -3.168<br>p = 0.006
News Format: Headline, Picture & Lede (vs. Headline)				0.106<br>z = 0.932<br>p = 0.353					0.610<br>z = 4.085<br>p = <0.001
News source: Source (vs. No source)					-0.219<br>z = -1.749<br>p = 0.082				-0.013<br>z = -0.174<br>p = 0.864
Accuracy Scale: 6 (vs. 4)						-0.410<br>z = -2.800<br>p = 0.006			0.459<br>z = 2.874<br>p = 0.011
Accuracy Scale: 7 (vs. 4)						-0.011<br>z = -0.060<br>p = 0.952			-1.006<br>z = -6.327<br>p = <0.001
Accuracy Scale: binary (vs. 4)						-0.367<br>z = -2.499<br>p = 0.013			-0.368<br>z = -14.317<br>p = <0.001
Accuracy Scale: other (vs. 4)						-0.145<br>z = -1.026<br>p = 0.306			
Symmetrie: perfect (vs. imperfect)							-0.505<br>z = -4.308<br>p = <0.001		-0.167<br>z = -1.295<br>p = 0.214
False news: verified by researchers (vs. taken from fact check sites)								0.096<br>z = 0.475<br>p = 0.635	
Num.Obs.	301	86	300	281	257	298	287	292	68
AIC	461.9	39.6	459.0	435.8	389.3	461.8	380.0	453.2	-7.3
BIC	476.7	49.4	477.5	454.0	403.5	487.6	394.7	467.9	19.3

Note: Results of meta-regressions for different moderator variables. No adjustements have been made. Note that the last column for the model with all moderators does not display effect sizes for all moderator categories, because not all combinations of moderator categories are present in the data, case in which no effect size can be computed.

Table B2

Moderator effects on Skepticism bias

	Country	Concordance	Family	Format	Source	Scale	Symmetrie	False news	All
intercept	0.295	-0.203	0.300	0.229	0.282	0.507	0.410	0.375	0.337
	z = 5.539	z = -1.929	z = 5.392	z = 4.454	z = 4.350	z = 4.749	z = 5.132	z = 8.967	z = 1.353
	p = <0.001	p = 0.064	p = <0.001	p = <0.001	p = <0.001	p = <0.001	p = <0.001	p = <0.001	p = 0.195
Country: US (vs. nonUS)	0.036								0.380
	z = 0.472								z = 1.895
	p = 0.638								p = 0.076
Political Concordance : Discordant (vs. Concordant)		0.779							0.844
		z = 10.043							z = 9.648
		p = <0.001							p = <0.001
News family: Other (vs. Covid)			-0.020						
			z = -0.222						
			p = 0.825						
News family: Political (vs. Covid)			0.035						
			z = 0.426						
			p = 0.671						
News Format: Headline & Picture (vs. Headline)				0.215					-1.253
				z = 2.449					z = -5.131
				p = 0.015					p = <0.001
News Format: Headline, Picture & Lede (vs. Headline)				0.328					-0.002
				z = 3.399					z = -0.010
				p = <0.001					p = 0.992
News source: Source (vs. No source)					0.115				0.076
					z = 1.304				z = 0.394
					p = 0.194				p = 0.699
Accuracy Scale: 6 (vs. 4)						-0.048			2.292
						z = -0.346			z = 9.379
						p = 0.730			p = <0.001
Accuracy Scale: 7 (vs. 4)						-0.496			-1.329
						z = -3.674			z = -3.955
						p = <0.001			p = 0.001
Accuracy Scale: binary (vs. 4)						-0.285			0.692
						z = -2.474			z = 13.283
						p = 0.014			p = <0.001
Accuracy Scale: other (vs. 4)						-0.153			
						z = -1.163			
						p = 0.246			
Symmetrie: perfect (vs. imperfect)							-0.152		-1.661
							z = -1.651		z = -12.725
							p = 0.101		p = <0.001
False news: verified by researchers (vs. taken from fact check sites)								-0.484	
								z = -4.851	
								p = <0.001	
Num.Obs.	301	86	300	281	257	298	287	292	68
AIC	506.5	103.7	508.3	441.3	439.2	491.0	484.2	481.4	73.5
BIC	521.3	113.5	526.9	459.5	453.4	516.9	498.9	496.1	100.2

Note: Results of meta-regressions for different moderator variables. No adjustements have been made. Note that the last column for the model with all moderators does not display effect sizes for all moderator categories, because not all combinations of moderator categories are present in the data, case in which no effect size can be computed.

Table B3

Frequency table of scales

	Imperfect Symmetry	Perfect symmetry	NA
Papers	24	39	4
Samples	58	124	13
Effects	80	209	14

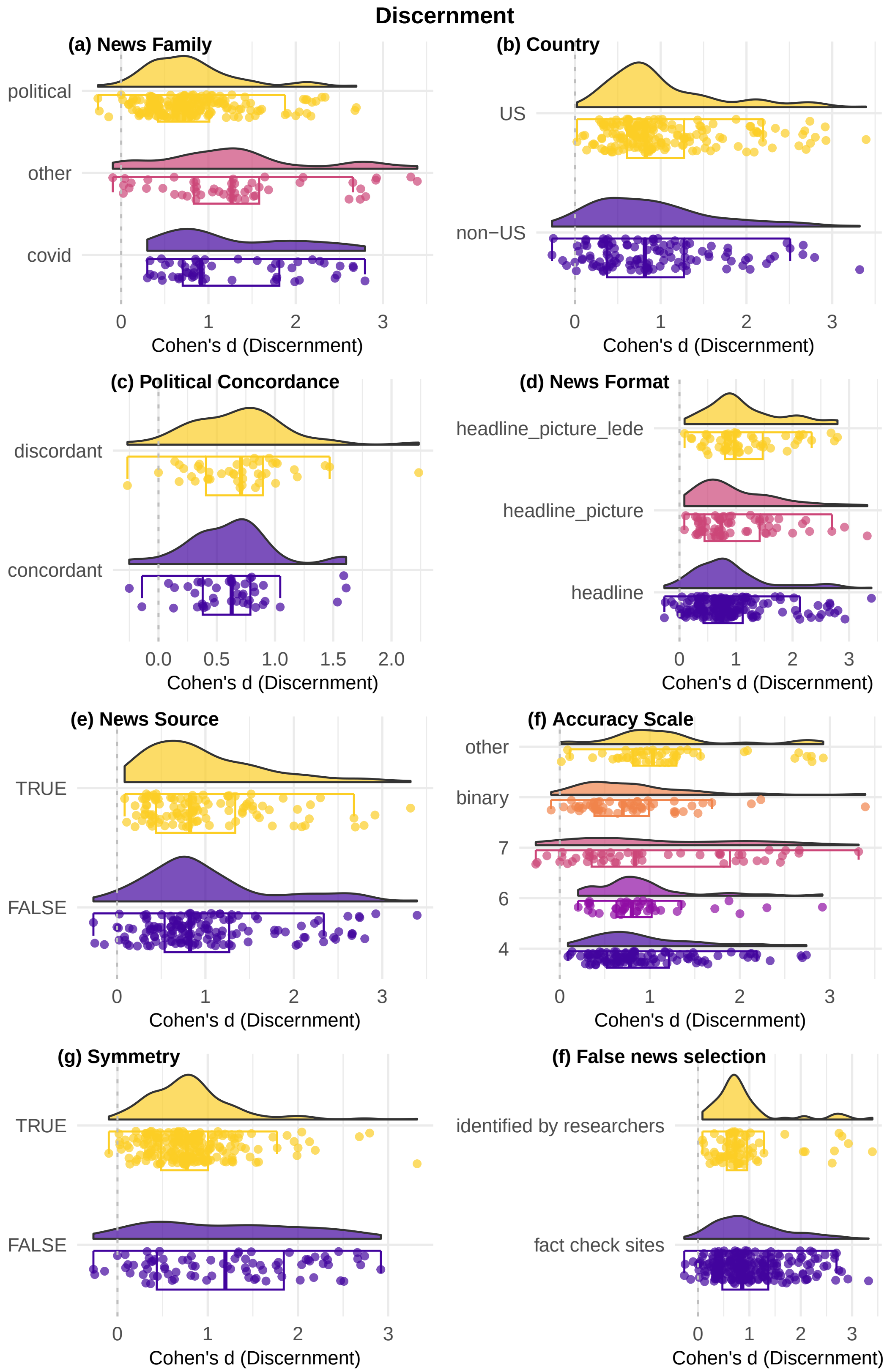

Figure B1. *Moderator effects on discernment.* The figure shows the distribution of effect sizes for discernment by moderator variables.

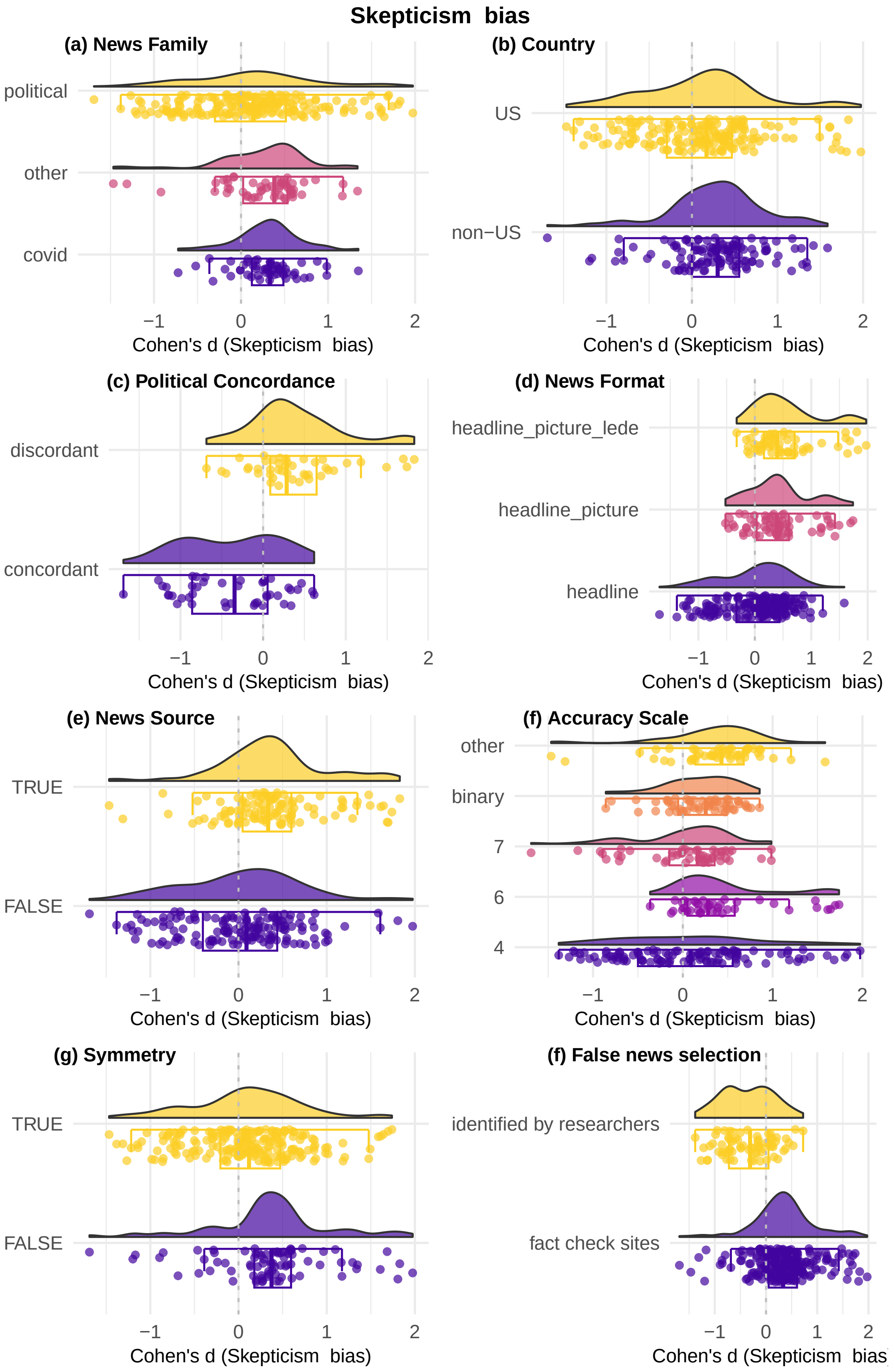

Figure B2. Moderator effects on skepticism bias. The figure shows the distribution of effect sizes for skepticism bias by moderator variables.

"Fake", is accurate "Yes" or "No", is accurate and unbiased "Yes" or "No"), and most Likert-scales (1 to 7: "Definitely fake" [1] to "Definitely real" [7], "Very unreliable" [1] to "Very reliable" [7], "Extremely unlikely" [1] to "Extremely likely" [7], "Extremely unbelievable" [1] to "Extremely believable" [7]; 1 to 6: "Extremely inaccurate" [1] to "Extremely accurate" [6], "Completely false" [1] to "Completely true" [6]). Yet, we coded the most common scale, a 4-point Likert scale ([1] not at all accurate, [2] not very accurate, [3] somewhat accurate, [4] very accurate), as not perfectly symmetrical. We coded two other Likert scales as not perfectly symmetrical ("not at all trustworthy" [1] to "very trustworthy" [10]; "not at all" [1] to "very" [7]).

Third, we investigated whether H1 and H2 hold for both perfectly symmetrical and imperfectly symmetrical scales. While both H1 and H2 hold for both symmetry types, we found that studies with perfectly symmetric scales tend to yield lower discernment scores (Δ Discernment = -0.51 [-0.74, -0.27]) than studies relying on scales that are at least slightly asymmetric (Baseline discernment slightly asymmetric scales = 1.39 [1.18, 1.6]). Importantly, we do not find a difference regarding skepticism bias.

The results suggest that imperfectly symmetrical scales may inflate discernment. However, the symmetry of response scales was not a factor that was experimentally manipulated, and the studies we compare in our model differ in many other ways and the observed difference is likely confounded.

Proportion of true news. Most studies exposed participants to 50% false and 50% true news, whereas outside of experimental settings, people on average are exposed to much more true news than false news[55]. This inflated proportion of false news may increase discernment or make participants more skeptical of true news. Experimental evidence suggests that the ratio of false news has no effect on discernment and slightly increases skepticism in news judgment[6]. Figure B3 shows effect sizes for discernment and skepticism bias as a function of news ratio. Due to the very uneven number of effect sizes, it does not seem reasonable to run a meta-regression to test this. However, Fig. B3 suggests no obvious trend with regard to the share of true news ratio. Besides, as for the other moderator variables, any observed association is likely to be confounded by other factors.

Selection of false news. The majority of studies selected false news items from fact checking sites (e.g. Snopes). However, in some studies, veracity of news items has been established by researchers (or fact-checkers hired by researchers). Table B4 lists these studies.

Three of these studies reduced researcher selection bias by automatically sampling news items[38,57,58]. We discuss these studies in detail in Appendix G. Here, we rely on the slightly broader definition of news items not taken from fact-checking websites. As shown in Tables B1 we find no difference in discernment when comparing studies that relied on news from fact-checking sites, compared to studies in which researchers established veracity of news items. We do find a difference regarding skepticism bias (see Table B2), such that studies relying on false news items as verified by the researchers show reduced (to almost 0) skepticism bias, compared to studies relying on false news items as verified by fact-checking organizations.

Note that, as with all between-study moderators, these estimates are likely confounded.

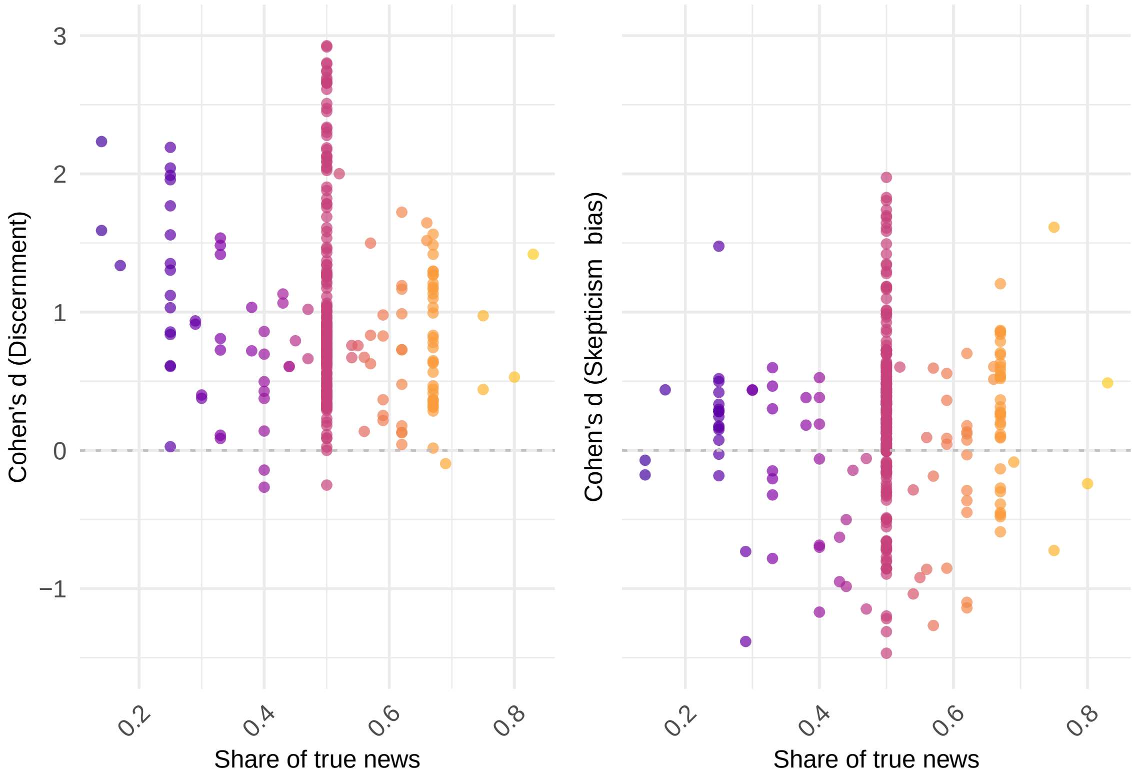

Figure B3. Effects of true-false ratio. Effect sizes plotted by their share of true news among all news that an individual participant saw.

The vast majority of effect sizes in the 'verified by researchers' category come from a single panel study[57]. This paper finds a negative skepticism bias for politically concordant news, suggesting that people are gullible towards information they politically approve. They did not find a skepticism bias for politically discordant items. Political concordance, therefore, is one reasonable candidate of a confounder for the observed difference regarding false news selection. However, as shown in Appendix G, the (comparatively few) effect sizes from two other two studies relying on automated news selection also consistently yield a negative skepticism bias (i.e. gullibility bias). Automated news selection might therefor be a relevant factor, perhaps more important than merely not selecting news from fact-checked websites (as did the other studies in Table B4).

Table B4
Studies that did not select false news items from fact-checking sites.

	Reference
1	Lutzke, L., Drummond, C., Slovic, P., & Árvai, J. (2019). Priming critical thinking: Simple interventions limit the influence of fake news about climate change on Facebook. Global Environmental Change, 58, 101964. https://doi.org/10.1016/j.gloenvcha.2019.101964
2	Roozenbeek, J., Maertens, R., Herzog, S. M., Geers, M., Kurvers, R., & Sultan, M. (2022). Susceptibility to misinformation is consistent across question framings and response modes and better explained by myside bias and partisanship than analytical thinking. Judgment and Decision Making, 17(3), 27.
3	Maertens, R., Götz, F. M., Schneider, C. R., Roozenbeek, J., Kerr, J. R., Stieger, S., McClanahan, W. P., Drabot, K., & Linden, S. van der. (2021). The Misinformation Susceptibility Test (MIST): A psychometrically validated measure of news veracity discernment [Preprint]. PsyArXiv. https://doi.org/10.31234/osf.io/gk68h
4	Gottlieb, J., Adida, C., & Moussa, R. (2022). Reducing Misinformation in a Polarized Context: Experimental Evidence from Côte d'Ivoire. OSF Preprints. https://doi.org/10.31219/osf.io/6x4wy
5	Kirill Bryanov, Reinhold Kliegl, Olessia Koltsova, Tetyana Lokot, Alex Miltsov, Sergei Pashakhin, Alexander Porshnev, Yadviga Sinyavskaya, Maksim Terpilovskii & Victoria Vziatysheva (2023) What Drives Perceptions of Foreign News Coverage Credibility? A Cross- National Experiment Including Kazakhstan, Russia, and Ukraine, Political Communication, 40:2, 115-146, DOI: 10.1080/10584609.2023.2172492
6	Altay, S., & Gilardi, F. (2023). People Are Skeptical of Headlines Labeled as AI-Generated, Even if True or Human-Made, Because They Assume Full AI Automation. OSF. https://doi.org/10.31234/osf.io/83k9r
7	Garrett, R. K., & Bond, R. M. (2021). Conservatives' susceptibility to political misperceptions. Science Advances, 7(23), eabf1234. https://doi.org/10.1126/sciadv.abf1234
8	Aslett, K., Sanderson, Z., Godel, W., Persily, N., Nagler, J., & Tucker, J. A. (2024). Online searches to evaluate misinformation can increase its perceived veracity. Nature, 625(7995), 548–556. https://doi.org/10.1038/s41586-023-06883-y
9	Allen, J., Arechar, A. A., Pennycook, G., & Rand, D. G. (2021). Scaling up fact-checking using the wisdom of crowds. Science Advances, 7(36), eabf4393.

Appendix C
Individual level data

We compare the results of our main meta model to the individual-level data with the following procedure: First, we restrict our data to (i) only studies using a non-binary response scale and (ii) only those studies that we have individual-level data on. This subset consists of 14 articles ($N_{Participants} = 17214$, $N_{Observations} = 354425$). Second, we run the same meta-analytic model as in the main analysis on the effect sizes of that subset of studies. Third, we take the individual-level data of that subset of studies and run a mixed model on it.

The meta-model estimates are standardized. To be able to compare results, we standardized participants' accuracy ratings in the individual-level data as follows: Within each sample, we calculated the standard deviation of accuracy ratings (false and true news combined). Then, for each sample, we divided accuracy ratings by the respective standard deviation.

We use the `lme4` package[76] and its `lmer()` function to run the mixed models. The mixed models include random effects by participant (each participant provides several ratings for both true and false news) and by sample for both the intercept and the effect of veracity. In our models, participants are nested in samples.

As shown in Fig. C1, this individual-level analysis yields an estimate very similar to our meta-analytic average.

How skilled were individual participants?

In our meta analysis, we find that people discern well between true and false news–on average. But how skilled are individual participants?

There are two ways to go about this: (i) How good were individual participants in discerning true from false?, and (ii) How good were individual participants in correctly judging the veracity of news?

As for the former, we have provided an answer in the main analysis (see Fig. **??**). Here, we report the absolute number of individuals with a positive vs. negative discernment and skepticism bias score in Table C1.

Table C1

	Discernment	Skepticism bias
negative	5385 (0.201)	10980 (0.409)
positive	21435 (0.799)	15840 (0.591)

Note. Frequency table of total number of participants that had a positive or negative score for both outcomes. Values in brackets indicate the share of participants for the respective outcome (i.e. column).

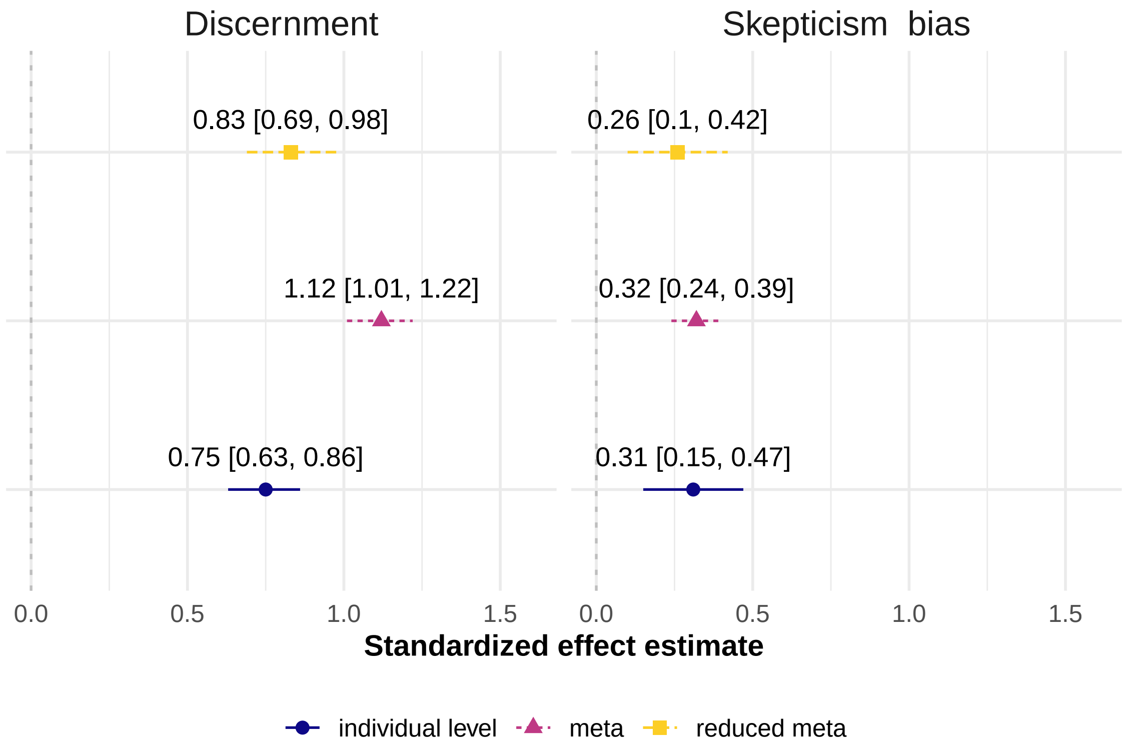

Figure C1. Comparison of meta to individual level analysis (continuous scales only). "Meta" corresponds to the main results reported in the main article, run on all n = 303 effect sizes; "meta reduced" are the same meta-analytic models as in the main analysis but run on the subset of 14 articles ($N_{Participants}$ = 17214, $N_{Observations}$ = 354425; making for 65 effect sizes) for which we have individual level data; "individual-level" corresponds to the result of mixed effect models run on the same subset of individual-level data. Symbols represent model estimates, horizontal bars 95% confidence intervals.

To answer the latter question, 'How good were individual participants in correctly judging the veracity of news?', we collapsed all likert scales into binary ones. For example, on a 4-point scale, we coded responses of 1 and 2 as not accurate (0) and 3 and 4 as accurate (1). For scales, with a mid-point (example 3 on a 5-point scale), we coded midpoint answers as NA. For each participant, we then identified the instances in which individuals classify news judgments correctly (i.e. false news as false and true news as true), and calculate the share of correct judgments among all judgments. For example, a participant rating one true news item as true and one fake news item as true has a share of correct judgments of 50%. Fig. C2 shows the cumulative percentage of participants for different shares of correct judgments.

Only 23.90 % of participants were at chance or worse in judging the veracity of news items. The better 50% of participants were correct at least 66.67 % of the time in their news judgments.

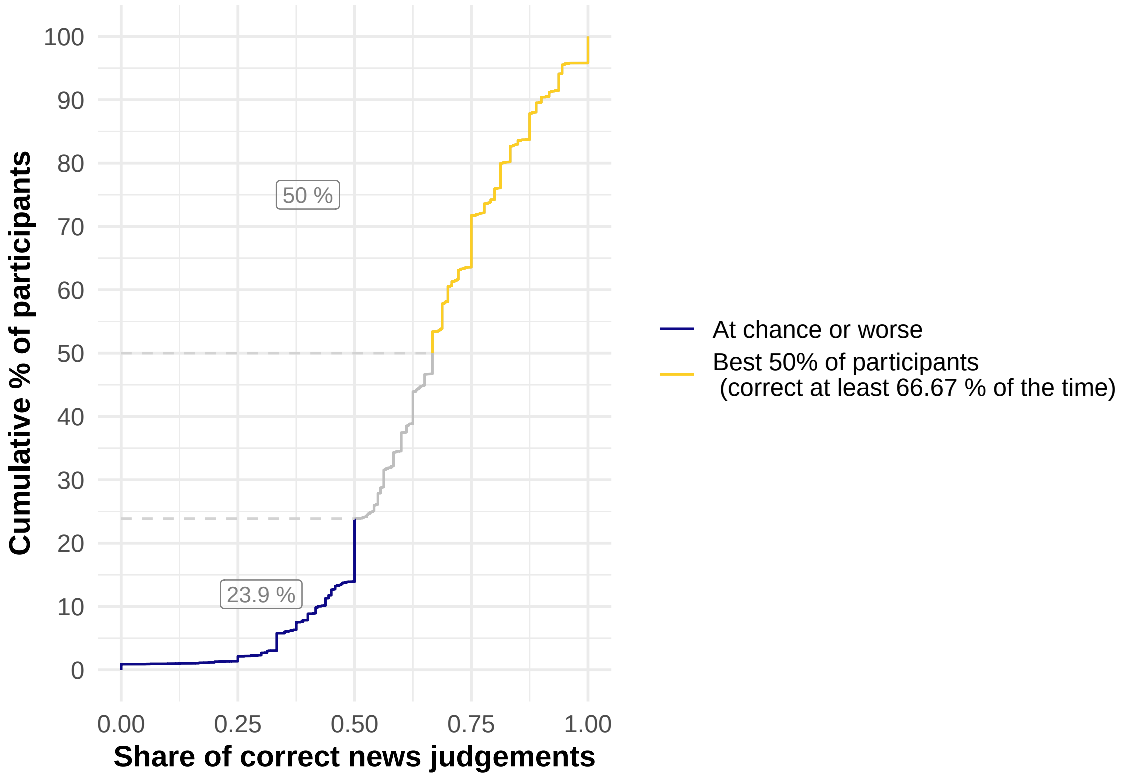

Figure C2. Cumulative distribution of participants as a function of the quality of their news judgments (i.e. the share of correct judgments among all judgments for each participant). To read the graph, pick a share of correct judgments on the X-axis, go vertically to the curve, from the curve go horizontally to the y-axis and read the share of participants who performed exactly this well or worse.

Note that before, we found that only 12.36 % of people had a negative discernment score. How is that compatible with 23.90 % of people performing at chance or worse?

That is because discernment and performing better than chance are distinct measurements. For example, people can be overall correct more than half of the time, but do considerably worse for true news than for fake news, yielding a negative discernment score. As shown in table C2, there are 2103 participants performing at chance or worse, but have a positive discernment score nevertheless (compared to only 526 participants with a negative discernment score who performed better than chance).

For a precise example taken from[77], see Table C3. The participant correctly identified the veracity of 8 out of 14 news items, i.e. performed better than chance. However, the participant rated only one true news item of five (correctly) as accurate. At the same time, the participant made two mistakes and classified two out of nine false news items as accurate. On average, the participant rated true news as less accurate ($1/5 = 18/90$) than false news ($2/9 = 20/90$), yielding a negative discernment score.

Table C2

Difference	n_subjects
better than chance but negative discernment	526
chance or worse but positive discernment	2103
same	24417

Note. Frequency table of participants, grouped by whether the direction of their score differs between discernment and share of correct judgements

Table C3

unique_participant_id	veracity	variable	value
Sultan_2022_1_90	fake	n_accurate	2.00
Sultan_2022_1_90	fake	mean_accurate	0.22
Sultan_2022_1_90	fake	n_correct	7.00
Sultan_2022_1_90	fake	n_ratings	9.00
Sultan_2022_1_90	true	n_accurate	1.00
Sultan_2022_1_90	true	mean_accurate	0.20
Sultan_2022_1_90	true	n_correct	1.00
Sultan_2022_1_90	true	n_ratings	5.00

Note. Example of a single participant who rated news items on a binary scale and obtained a negative discernment score while performing better than chance.

Appendix D
Binary vs. continuous scales

Do people answer differently on binary scales than on non-binary scales? Our moderator analysis suggest that studies with binary scales yield both (i) lower discernment and (ii) less skepticism bias. In this section, we first check if we observe this difference more generally between all Likert scales (i.e. not only the 4-point scale used as reference in our moderator analysis), and binary scales. We find a statistically significant difference regarding discernment, but not regarding skepticism bias. As discussed in the moderator analysis, these observations might be confounded by all sorts of factors by which studies differ. Here, we focus on whether they could be the result of a measurement problem: What difference does it make to record responses on a binary scale, compared to a Likert scale? In a first step, to provide a test, we use a subset of studies we have individual-level data on, and collapse Likert scale response into dichotomous answers. For example, on a 4-point scale, we coded responses of 1 and 2 as not accurate (0) and 3 and 4 as accurate (1). For scales with a mid-point (example 3 on a 5-point scale), we coded midpoint answers as 'NA'. We find a skepticism bias with the original Likert scale version (see also Appendix C), but not with the dichotomous version. In a second step, we look at studies we have individual-level data on and which use binary answer scales. For these studies, we do find both positive discernment and positive skepticism bias, although smaller estimates than our overall meta-analytic averages. We replicate this finding when adding the dichotomized version of the Likert scale studies from the first test. We further show that these results hold when using more appropriate summary statistics for binary outcomes, namely (log) odds ratios. Taken together, this suggests that skepticism bias stems partly from mis-classifications (the observed skepticism bias in binary response studies), but partly from degrees of confidence (the difference between the Likert scale version and the collapsed binary scale version). Across all analyses presented in this section, we conclude that people tend to (i) classify true news as false more often than false news as true and (ii) even when classifying equally well for both and true news, they rate true news as less extremely accurate than false news as inaccurate, suggesting lower confidence in their answers for true news.

Meta-regression

We ran a meta-regression using scale type (two levels: binary vs. continuous) as a predictor variable. Table D1 summarizes the results, and Fig. D1 illustrates them. The analysis suggests that discernment (but not skepticism bias) is more enhanced among continuous studies.

Dichotomizing likert scale responses

To further investigate the effect of scale type, we run a test on a subset of studies that we have individual-level data on and that used Likert scales. For this subset, we made two versions: (i) a version with the original Likert scale scores; (ii) a dichotomized version where we collapsed the Likert scale scores into either 'false' or 'true'. For example, on a 4-point scale, we coded responses of 1 and 2 as not accurate (0) and 3 and 4 as accurate (1). For scales, with a mid-point (example 3 on a 5-point scale), we coded midpoint answers as 'NA'.

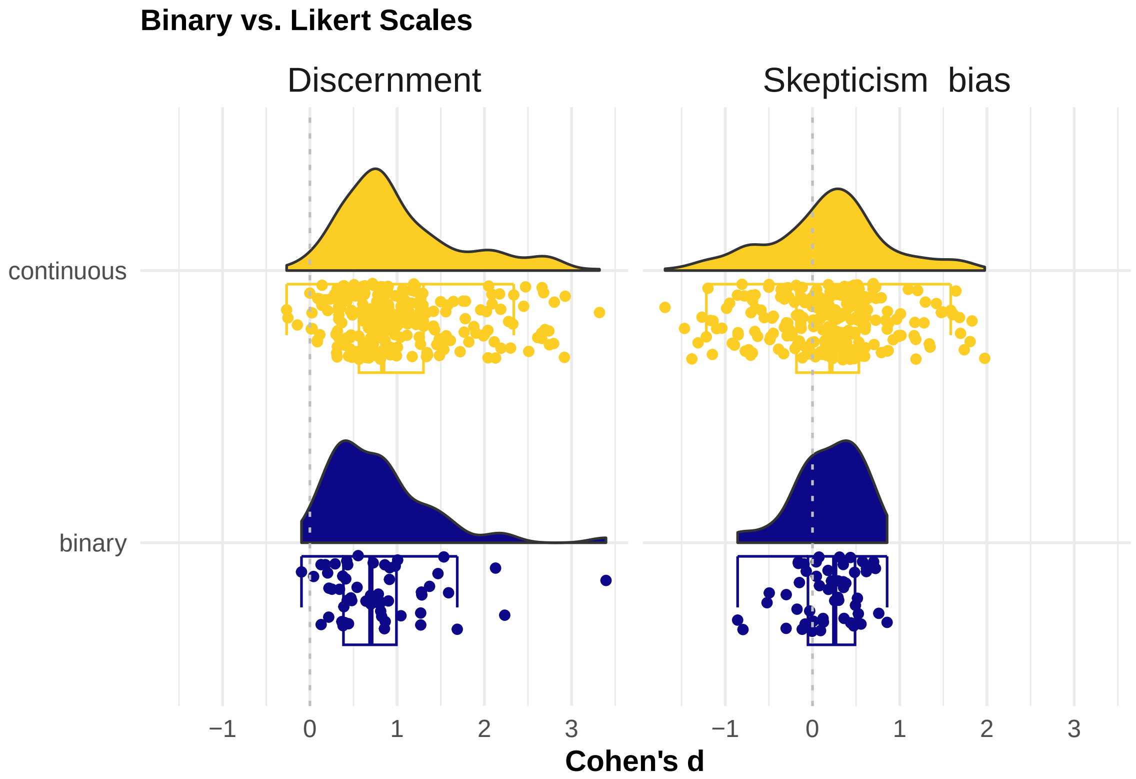

Figure D1. Distribution of effect sizes (Cohen's d) grouped by whether a binary or continuous (Likert-scale type) response scale was used.

Table D1

Binary vs. Likert Scales

	Discernment	Skepticism bias
intercept	0.921	0.221
	z = 9.305	z = 5.115
	p = <0.001	p = <0.001
Continuous (vs. binary)	0.240	0.117
	z = 2.246	z = 1.795
	p = 0.026	p = 0.074
Num.Obs.	301	301
AIC	462.2	505.2
BIC	477.0	520.0

Note: Results of a meta-regression using scale type (two levels: binary vs. continuous) as the moderator variable. No adjustements have been made.

Table D2
Original Likert-scale vs. dichotomized version

	Original Likert scale		Dichotomized	
	Discernment	Skepticism bias	Discernment	Skepticism bias
Estimate	0.835	0.284	0.766	0.070
	z = 11.768	z = 3.894	z = 11.496	z = 1.164
	p = <0.001	p = <0.001	p = <0.001	p = 0.244
Num.Obs.	35	35	35	35
AIC	45.7	47.6	41.1	34.1
BIC	50.3	52.3	45.8	38.7

Note: Meta-analyses following the modeles of the main paper, on the subset of studies that we have individual-level data on and that used Likert scales. For this subset, we made two versions: (i) a version with the original Likert scale scores; (ii) a dichotomized version where we collapsed the Likert scale scores into either 'false' or 'true'. For example, on a 4-point scale, we coded responses of 1 and 2 as not accurate (0) and 3 and 4 as accurate(1). For scales, with a mid-point (example 3 on a 5-point scale), we coded midpoint answers as 'NA'. No adjustments have been made.

We calculate the summary statistics for both versions and run the same meta-analytic models on that subset. Table D2 summarizes the results.

Binary response scales

Above, we found that skepticism bias disappears when dichotomizing scales of studies intially recording responses on Likert scales. How about studies who recorded responses on a binary scale? Here, we focus on the subset of studies that we have raw, individual-level data on, and focus on the studies that used a binary response scale.

In addition the the main meta-analytic models, here, we additionally present results based on more appropriate effect sizes for binary data, namely log odds ratios (logORs). In our main analysis, we combined studies which measure perceived accuracy on a continuous scale, and studies who do so on a binary scale. This is not problematic per se–there are statistical methods to compare effects on both scales[32]. These require, however, appropriate summary statistics for both scales. For continuous measures, means and standard deviations are fine; for binary measures we would need, for example, odds or risk ratios. The problem we were facing is that authors did not provide the appropriate summary statistics for binary scales. Instead, they tended to report means and standard deviations, just as they do for continuous outcomes. For the main analysis, we made the decision to treat continuous and binary scales in the same way, glossing over potential biases from inappropriate summary statistics.

Table D3

Veracity	Rated as accurate	Rated as not accurate	Sum
fake	29538 (0.292)	71622 (0.708)	101160 (1)
true	57889 (0.611)	36818 (0.389)	94707 (1)

Note. Frequency of responses (among individual-level studies with binary response scales)

Odds ratios. We first calculated the odds ratios from the raw data[1]. The 'odds' refer to the ratio of the probability that a particular event will occur to the probability that it will not occur, and can be any number between zero and infinity[32]. It is commonly expressed as a ratio of two integers. For example, in a clinical context, 1 out of 100 patients might die; then the odds of dying are '0.01', or '1:100'.

The odds *ratio* (OR) is the ratio of the Odds. The odds ratio that characterizes discernment is calculated as

$$OR_{Accuracy} = \frac{(Accurate_{true}/NotAccurate_{true})}{(Accurate_{false}/NotAccurate_{false})}$$

If the OR is `1`, participants were just as likely to rate items as 'accurate' when looking at true news as they were when looking at false news. If the OR is `> 1`, then participants rated true news as more accurate than fake news. An OR of `2` means that participants were twice as likely to rate true news as accurate compared to false news.

The OR for skepticism bias is calculated as

$$OR_{Error} = \frac{(NotAccurate_{true}/Accurate_{true})}{(Accurate_{false}/NotAccurate_{false})} = \frac{\frac{1}{(NotAccurate_{true}/Accurate_{true})}}{(Accurate_{false}/NotAccurate_{false})} = \frac{1}{OR_{Accuracy}}$$

For our analysis, we calculated the odds ratio (OR) for both accuracy and error. More precisely, we expressed the OR on a logarithmic scale, also referred to as "log odds ratio"(logOR). As for odds ratios, if the log odds ratio is positive, it indicates positive discernment/skepticism bias[2].

Table D3 shows the frequency of answers by veracity.

Meta-analyses. We ran two meta-analyses on two different data sets: The first data set consists of only studies that we have individual level data for and that used binary response scales. Results are displayed in Table D4. For reference, we also report a

[1] A general overview of appropriate summary statistics for binary outcomes can be found here[32]: https://training.cochrane.org/handbook/current/chapter-06#section-6-4)

[2] To interpret the magnitude of that difference we have to transform the logarithmic estimate back to a normal odds ratio. The reason we use the log odds ratios in the first place is that which makes outcome measures symmetric around 0 and results in corresponding sampling distributions that are closer to normality[69]

Table D4

Individual-level studies with binary response scale

	(based on individual data) Log OR		(based on meta data) Cohen's d		Mean change	
	Accuracy	Error	Accuracy	Error	Accuracy	Error
Estimate	1.256	0.464	0.654	0.239	0.296	0.110
	z = 9.531	z = 4.485	z = 9.206	z = 3.369	z = 10.324	z = 3.371
	p = <0.001	p = <0.001	p = <0.001	p = <0.001	p = <0.001	p = <0.001
Num.Obs.	19	19	32	32	32	32
AIC	40.6	31.0	-2.7	39.6	-59.2	-12.0
BIC	43.5	33.8	1.7	44.0	-54.8	-7.6

Note: Results of a meta-analyses using different effect-size estimators, on the subset of studies that we have individual-level data on and that use binary response scales. Note that the number of observations differ, because some samples provide several effect sizes in the meta-data. For the odds ratios based on the individual data, however, we calculated only one average effect size per sample. No adjustments have been made.

non-standardized estimator that likewise accounts for dependence between false and true news, namely the mean change (MC)[3]. The second data set consists of all studies that we have individual level data for, with ratings of those studies that originally used Likert-scale responses collapsed to binary outcomes (results in Table D5). In both analyses, we find (i) positive discernment and (ii) positive response bias, using both the same Cohen's d effect sizes of our main analysis and effect sizes expressed in log Odds Ratios. Note, however, that these estimates are smaller than the our overall meta-analytic averages.

[3]We use the term mean change in line with vocabulary used by the metafor package and its `escalc()` function that we use for all effect size calculations. It is in fact a simple mean difference but one that accounts for the correlation between true and false news in the calculation of the standard error (see[32]). Here is a direct link to the relevant chapter online: https://training.cochrane.org/handbook/current/chapter-23#section-23-2-7-1

Table D5

Individual-level studies with binary response scales and Likert scale ratings collapsed to binary responses

	Log OR		Cohen's d	
	Discernment	Skepticism bias	Discernment	Skepticism bias
Estimate	1.414	0.277	0.719	0.124
	z = 15.249	z = 3.036	z = 14.386	z = 2.858
	p = <0.001	p = 0.002	p = <0.001	p = 0.004
Num.Obs.	55	55	55	55
AIC	123.1	121.5	53.9	38.4
BIC	129.2	127.5	59.9	44.4

Note: An extension to the previous table, where in addition to the studies that used a binary response scale, we added a dichotomized response version for studies that used Likert scales.

Appendix E
Publication bias

To quantify asymmetry as visualized by the funnel plot, we ran Egger's regression test[78] following our pre-registration. The results are displayed in Table E1. The outcome variable in the Egger's regression test is the observed effect size divided by its standard error. The resulting value is a z-score, which tells us directly if an effect size is significant: If $z \geq 1.96$ or $z \leq -1.96$, we know that the effect is significant ($p < 0.05$). This outcome is regressed on the inverse of its standard error, a measure of precision, with higher values indicating higher precision[71]. The coefficient of interest in the Egger's test is the intercept, i.e. the estimated z-score when precision (the predictor variable) is zero. Given a precision of 0, or an infinitely large standard error, we would expect a z-score scattered around 0. However, when the funnel plot is asymmetric, for example due to publication bias, we expect that small studies with very high effect sizes will be considerably over-represented in our data, leading to a surprisingly high number of low-precision studies with high z-values. Due to this distortion, the predicted value of y for zero precision will be considerably larger than zero, resulting in a significant intercept. However, just as asymmetries in the funnel plot can stem from sources of heterogeneity other than publication bias, a positive Egger's regression is not proof for publication bias. In fact, because we had no a priori suspicion of publication bias–our outcomes have not been of the outcomes of interest in the original studies–we do not take the results of the Egger's test as indicative of publication bias.

Table E1
Egger's regression

	Discernment	Skepticism bias
(Intercept)	45.030	5.172
	t = 11.700	t = 1.391
	p = <0.001	p = 0.165
Inverse SE	0.114	0.150
	t = 2.222	t = 3.267
	p = 0.027	p = 0.001
Num.Obs.	303	303
R2	0.016	0.034
R2 Adj.	0.013	0.031
AIC	3103.1	3055.4
BIC	3114.3	3066.5
Log.Lik.	-1548.563	-1524.696
RMSE	40.12	37.08

Note: Results of Egger's regression test. No adjustments have been made.

Appendix F
Country comparison

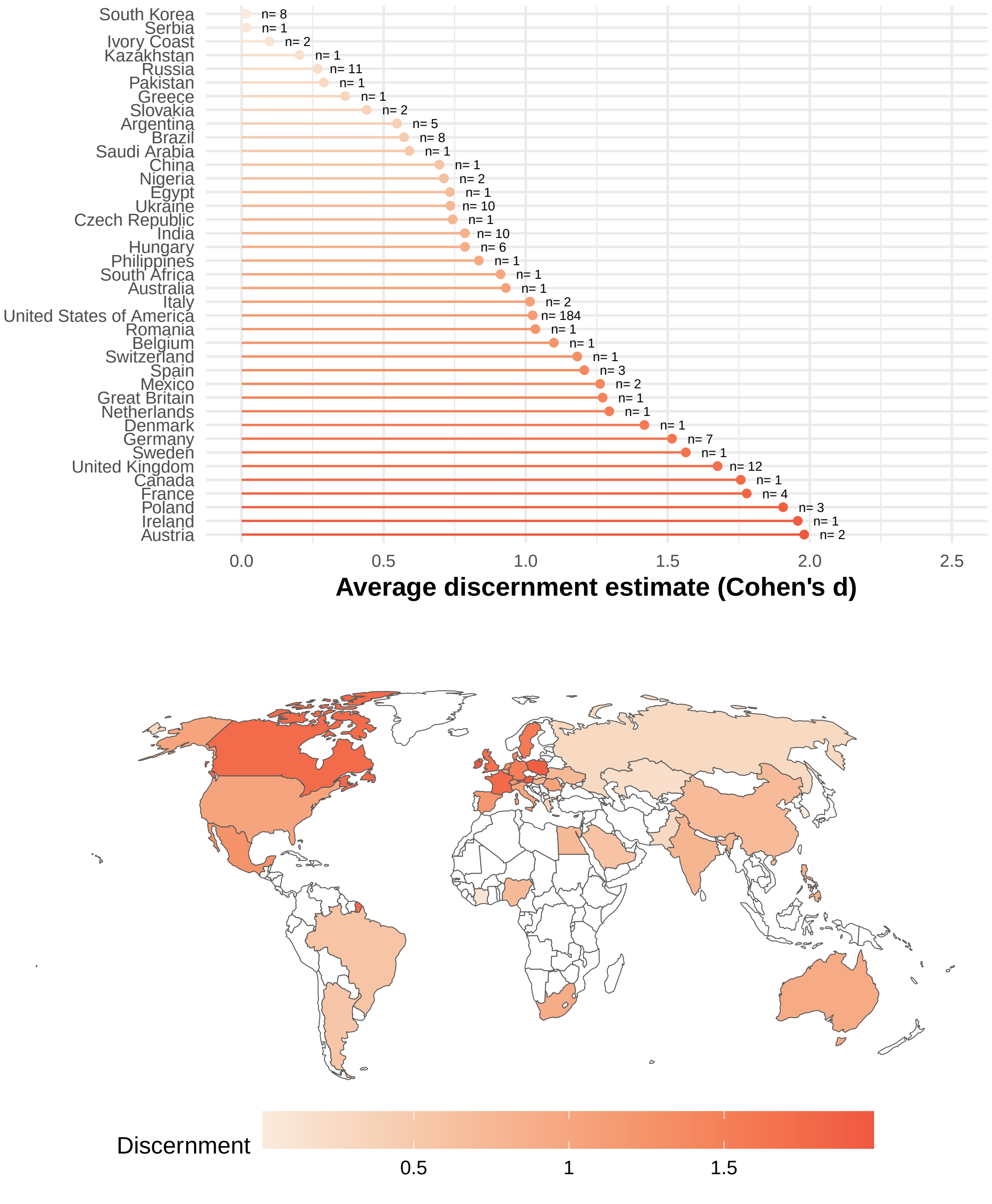

Figure F1. Discernment estimates by country.

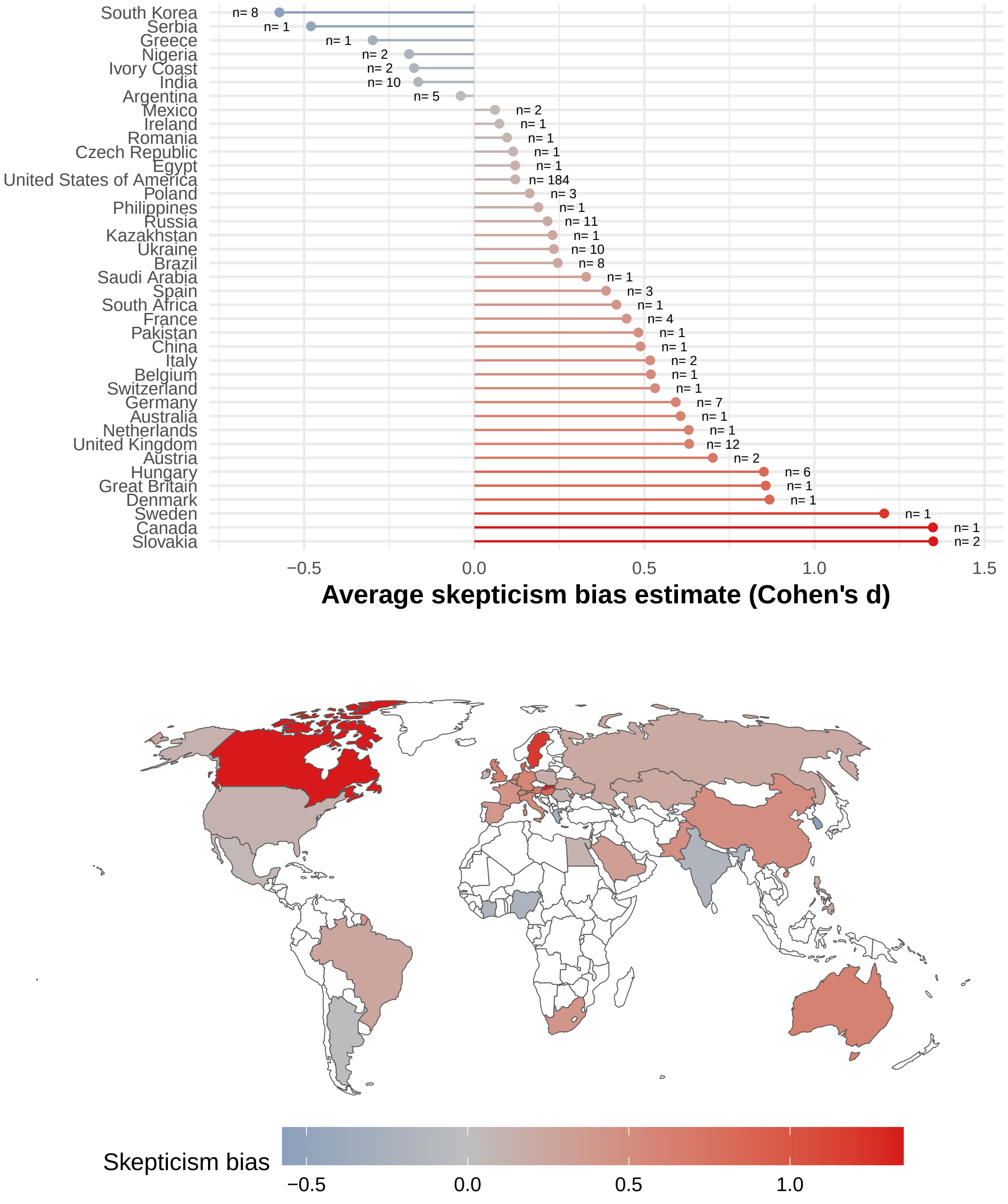

Figure F2. Skepticism bias estimates by country.

Appendix G
Selection bias

Skepticism bias could be an artifact of biased news selection for experiments. For example, one might suspect researchers to pick easy-to-detect false news and/or hard-to-detect true news (e.g. to avoid ceiling effects), thus inflating participants' skepticism of true news.

We believe that if there is such a bias, it is likely most relevant for false news. That is because we observe similar average accuracy ratings for true news in three studies (one of which included in our meta-analysis, namely[57]) that randomly sampled true news from high-quality mainstream news sites. These samples of headlines are free of any selection bias that may originate from researchers selecting not obviously accurate true headlines.[79] used CrowdTangle to automatically scrap 500 headlines from 20 mainstream news sites and had participants rate the accuracy of these headlines. The mean accuracy rating of these headlines was 5.05 (sd = 0.56) on a 7-point scale, or 0.68 if we transpose the scale to reach from 0 to 1. This is similar to our (unweighed) average true news rating (0.60) when scaling effect sizes to range from 0 to 1 (see Fig. **??**). Similarly,[80] automatically scraped true headlines using the Google News API. On a 7-point scale, the average true news rating was 4.45 (sd = 1.66), or 0.57 on a scale from 0 to 1. In a panel study over six months,[57] used the NewsWhip API to automatically scrap timely news headlines, selecting the most popular ones on social media. On a 4-point scale, the average true news rating was 2.99 (sd = 0.77), or 0.66 on a scale from 0 to 1. However, note that a study in a Russian news context finds lower accuracy ratings for true news than the average in our meta-analysis:[81] used web scraping to automatically download top news stories on politics and international news from Yandex News (Russia's largest news aggregator). Across the two studies, true news stories selected with this process were rated as true only 48% of the time (mean on binary scale = 0.48, sd = 0.50).

If not for true news, it seems likely that our results are affected by a selection bias for false news. Three studies included in our meta-analysis[38,57,58] automated their news selection by scraping headlines from media outlets. Fact-checkers hired by the researchers (or the researchers themselves, in the case of[57]) would establish their veracity. These studies are less biased in their news selection, and let participants rate news in real time (i.e. when news arguably matter most to people). As shown in Figure G1, the effect sizes extracted from these studies show that participants, on average, discerned between true and false headlines, but that they were better at rating true headlines as true than false headlines as false (suggesting a negative skepticism bias, i.e. a credulity bias).

One explanation of the discrepancies between the findings of[57],[58] and[38] on the one side, and the findings of our meta-analysis on the other, is that fact-checking websites pick more easy-to-fact-check misinformation. In that case, many false news included in the three studies would have never appeared on fact-checking websites, and are therefor quite different from the selection of false news in other studies included in our meta-analysis[4]. But note that, although plausible, it is not clear whether the observed discrepancies are in fact driven by the selection of false news. For example, in the case of[57], a reasonable candidate

[4]It is unlikely that this difference is due to timeliness of the three studies:[58] found that participants were better at decking false news within 48 hours of publication compared to 3 months or more after.

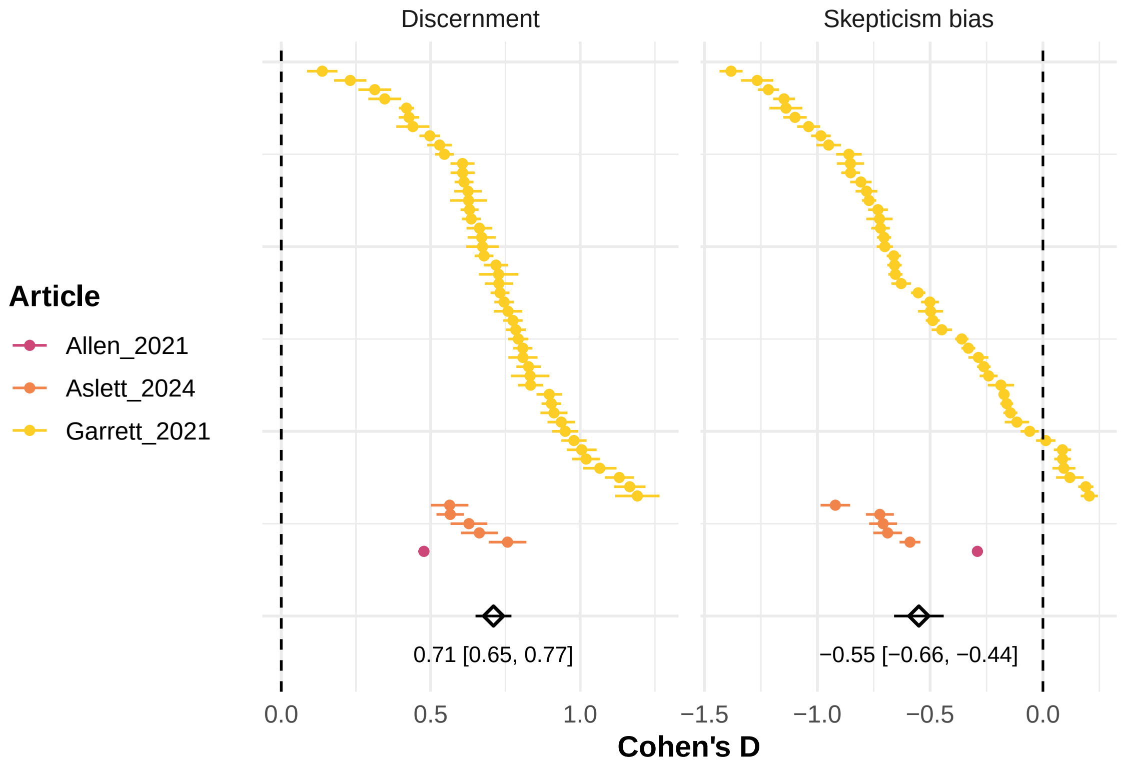

Figure G1. Forest plots for discernment and skepticism bias, for the three studies using automated news selection. Effects are weighed by their sample size. Effect sizes are calculated as Cohen's d. Horizontal bars represent 95% confidence intervals. The average estimate (black diamond shape at the bottom of the figure) is the result of a multilevel meta model with clustered standard errors at the sample level.

for a confounder is political concordance (see below). In their large panel study included in our meta-analysis,[57] relied on automatically scraped popular headlines and classified coded their political concordance. As shown in table G1, a moderator analysis suggests that the overall negative skepticism bias (i.e. the credulity bias) is at least partially driven by political concordance. Contrary to the findings in our meta-analysis (including data from[57]), their participants showed a strong tendency towards credulity when news headlines were concordant with their political stance, while only being slightly credulous when facing politically discordant headlines.

Table G1

Model results

	Garrett & Bond, 2021				Main results	
	Discernment	Skepticism bias	Discernment	Skepticism bias	Discernment	Skepticism bias
Estimate (intercept)	0.722	-0.539	0.657	-0.937	1.116	0.315
	z = 21.072	z = -8.681	z = 99.232	z = -133.087	z = 20.794	z = 8.109
	p = <0.001	p = <0.001	p = <0.001	p = <0.001	p = <0.001	p = <0.001
Political Concordance : Discordant (vs. Concordant)			0.148	0.897		
			z = 15.502	z = 95.491		
			p = <0.001	p = <0.001		
Num.Obs.	46	46	22	22	302	302
AIC	2.3	58.1	1761.6	2033.3	464.4	504.7
BIC	7.8	63.6	1763.7	2035.4	475.6	515.8

Note: Results from a meta-analysis of the panel study by Garrett & Bond 2021. The results for the moderator analysis for political concordance are based on less observations than the overall analysis, because the latter includes politically neutral headlines and participants who did identify as neither democrat nor republican. For reference, we included the main results from the meta-analysis (including the study by Garrett and Bond). No adjustments have been made.

Appendix H
Signal Detection Theory

Our two measures–discernment and skepticism bias–are akin to two measures of Signal Detection Theory (SDT): d' (sensitivity), and c (response bias). As our discernment measure, a positive d' score indicates that people rate true news as more accurate than false news. As our skepticism bias measure, a positive c score arises when the miss rate (rating true news as not accurate) is greater than the false alarm rate (rating false news as accurate). A body of recent studies uses a SDT framework to evaluate people's news judgments[28,53,82]. Do the results from our measures align with those from an SDT framework?

As with all individual-level analysis before, we rely on the subset of individual-level data, which captures all instances of news ratings for all participants. If not already on a binary scale, we collapse Likert scale responses to a binary scale. This allows to us to calculate a d' and a c score for each participant. We apply corrections to avoid infinitely small or large outcome scores, following[82] and[28].

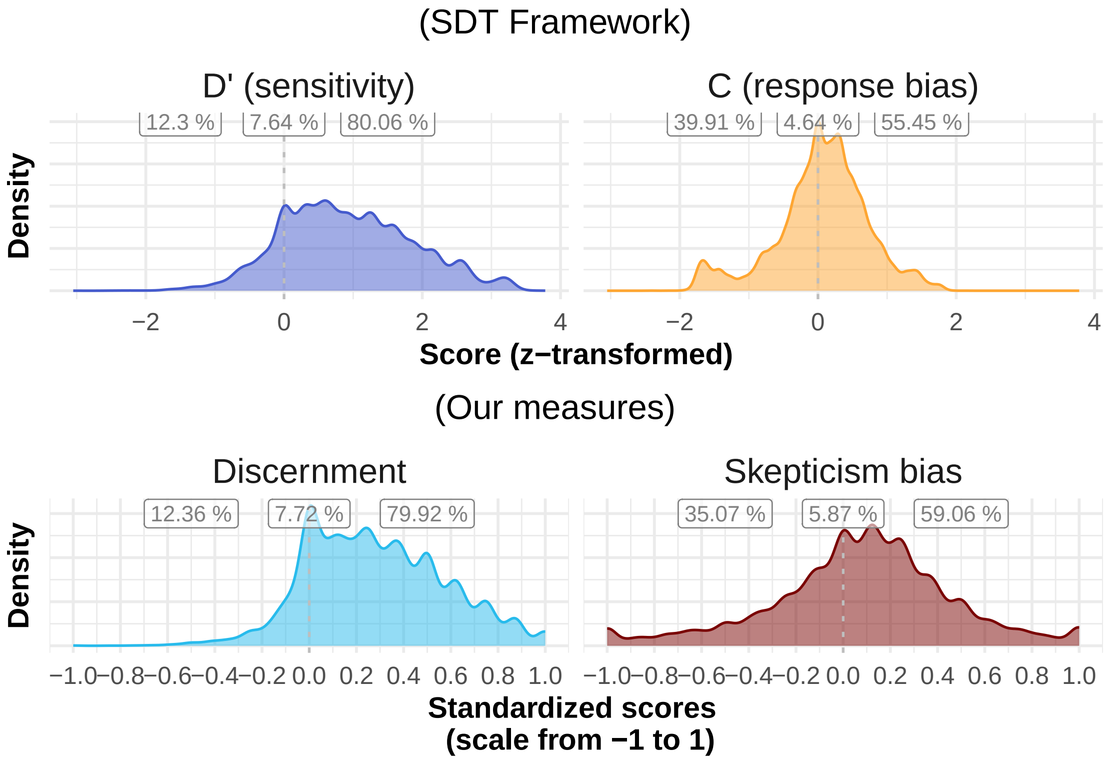

Figure H1. Distributions of outcomes of individual participants in the subset of studies that we have raw data on. The upper plot shows the distribution for the SDT outcome measures ("D prime", sensitivity, and "C", response bias). The lower plot corresponds to Fig. **??** from the results section of the main article and shows the distribution for our outcome measures for the same sample of participants (discernment and skepticism bias). The percentage labels (from left to right) represent the share of participants with a negative score, a score of exactly 0, and a positive score, for all measures respectively.

Fig. H1 visualizes the results. From descriptively comparing the share of participants with positive, negative, and scores of 0, we can see that sensitivity (D') and discernment yield almost identical results, while our skepticism bias measure qualifies slightly more people as having a tendency to be skeptical than the response bias C. However, conclusions remain the same.

Appendix I
Detailed search strings

First database search

For our initial database search, we used the following search strings:

- Scopus: ‘“false news” OR “fake news” OR “false stor*” AND “accuracy” OR “discernment” OR “credibilit*” OR “belief” OR “susceptib*”’

  Given the initially high volume of papers (12425), we added restrictions to only include articles that were likely (i) experimental, (ii) and exposed participants to both true and false news (addition to search string: ‘AND (LIMIT-TO (LANGUAGE , “English”)) AND (LIMIT-TO (DOCTYPE , “ar”) OR LIMIT-TO (DOCTYPE , “cp”)) AND (EXCLUDE (SUBJAREA , “PHYS”) OR EXCLUDE (SUBJAREA , “MATE”) OR EXCLUDE (SUBJAREA , “BIOC”) OR EXCLUDE (SUBJAREA , “ENER”) OR EXCLUDE (SUBJAREA , “IMMU”) OR EXCLUDE (SUBJAREA , “AGRI”) OR EXCLUDE (SUBJAREA , “PHAR”) OR EXCLUDE (SUBJAREA , “HEAL”) OR EXCLUDE (SUBJAREA , “EART”) OR EXCLUDE (SUBJAREA , “NURS”) OR EXCLUDE (SUBJAREA , “CHEM”) OR EXCLUDE (SUBJAREA , “CENG”) OR EXCLUDE (SUBJAREA , “VETE”) OR EXCLUDE (SUBJAREA , “DENT”)) AND (EXCLUDE (SUBJAREA , “COMP”) OR EXCLUDE (SUBJAREA , “ENGI”) OR EXCLUDE (SUBJAREA , “MATH”) OR EXCLUDE (SUBJAREA , “MEDI”)’)

- Google Scholar: ‘“Fake news” | “False news”|“False stor*” “Accuracy” | “Discernment”|“Credibility”|“Belief”|“Suceptib*”, no citations, no patents’

Second database search

For our second database search during revisions, we used the following search strings:

- Scopus: ’TITLE-ABS-KEY ((“false news” OR “fake news” OR “false stor” *OR “misinformation” OR “disinformation”) AND (“accuracy” OR “discernment” OR “credibilit”* OR “belief” OR “suceptib” *OR “reliab”* OR “vulnerabi*”)) AND (EXCLUDE (SUBJAREA , “DENT”) OR EXCLUDE (SUBJAREA , “CHEM”) OR EXCLUDE (SUBJAREA , “VETE”) OR EXCLUDE (SUBJAREA , “CENG”) OR EXCLUDE (SUBJAREA , “EART”) OR EXCLUDE (SUBJAREA , “AGRI”) OR EXCLUDE (SUBJAREA , “PHAR”) OR EXCLUDE (SUBJAREA , “MATH”) OR EXCLUDE (SUBJAREA , “ENGI”) OR EXCLUDE (SUBJAREA , “MEDI”) OR EXCLUDE (SUBJAREA , “NURS”) OR EXCLUDE (SUBJAREA , “HEAL”) OR EXCLUDE (SUBJAREA , “IMMU”) OR EXCLUDE (SUBJAREA , “BIOC”) OR EXCLUDE (SUBJAREA , “MATE”) OR EXCLUDE (SUBJAREA , “PHYS”) OR EXCLUDE (SUBJAREA , “ECON”) OR EXCLUDE (SUBJAREA , “ENER”) OR EXCLUDE (SUBJAREA , “COMP”)) AND (LIMIT-TO (DOCTYPE , “ar”) OR LIMIT-TO (DOCTYPE , “ch”) OR LIMIT-TO (DOCTYPE , “cp”)) AND (LIMIT-TO (LANGUAGE , “English”))’

- Google Scholar: ’“false news” OR “fake news” OR “false stor” *OR ”misinformation”*

OR "disinformation") AND ("accuracy" OR "discernment" OR "credibilit" OR "belief" OR "suceptib*" OR "reliab*" OR "vulnerabi*") , no patents'

Appendix J
Included studies

Table J1
Articles included in the meta analysis

id	reference	country	Effect Sizes	Participants
**1**	Ali, A., & Qazi, I. A. (2022). Digital Literacy and Vulnerability to Misinformation: Evidence from Facebook Users in Pakistan. Journal of Quantitative Description: Digital Media, 2.	Pakistan	1	674
**2**	Allen, J., Arechar, A. A., Pennycook, G., & Rand, D. G. (2021). Scaling up fact-checking using the wisdom of crowds. Science Advances, 7(36), eabf4393.	United States	1	1128
**3**	Altay, S., & Gilardi, F. (2023). People Are Skeptical of Headlines Labeled as AI-Generated, Even if True or Human-Made, Because They Assume Full AI Automation. OSF. https://doi.org/10.31234/osf.io/83k9r	United States	1	198
**4**	Altay, S., De Angelis, A., & Hoes, E. (2024). Media literacy tips promoting reliable news improve discernment and enhance trust in traditional media. Communications Psychology, 2(1), 1–9. https://doi.org/10.1038/s44271-024-00121-5	United States	3	984
**5**	Altay, S., Lyons, B. A., & Modirrousta-Galian, A. (2024). Exposure to Higher Rates of False News Erodes Media Trust and Fuels Overconfidence. Mass Communication and Society, 1–25. https://doi.org/10.1080/15205436.2024.2382776	United States	9	2836
**6**	Altay, S., Nielsen, R. K., & Fletcher, R. (2022). The impact of news media and digital platform use on awareness of and belief in COVID-19 misinformation [Preprint]. PsyArXiv. https://doi.org/10.31234/osf.io/7tm3s	Brazil, India, UK	9	6126
**7**	Altay, S., de Araujo, E., & Mercier, H. (2022). “If This account is True, It is Most Enormously Wonderful”: Interestingness-If-True and the Sharing of True and False News. Digital Journalism, 10(3), 373–394. https://doi.org/10.1080/21670811.2021.1941163	United States	3	897
**8**	Arechar, A. A., Allen, J., Berinsky, A. J., Cole, R., Epstein, Z., Garimella, K., Gully, A., Lu, J. G., Ross, R. M., Stagnaro, M. N., Zhang, Y., Pennycook, G., & Rand, D. G. (2023). Understanding and combatting misinformation across 16 countries on six continents. Nature Human Behaviour, 7(9), 1502–1513. https://doi.org/10.1038/s41562-023-01641-6	Argentina, Australia, Brazil, China, Egypt, India, Italy, Mexico, Nigeria, Philippines, Russia, Saudi Arabia, South Africa, Spain, UK, United States	16	8371

**9**	Aslett, K., Sanderson, Z., Godel, W., Persily, N., Nagler, J., & Tucker, J. A. (2024). Online searches to evaluate misinformation can increase its perceived veracity. Nature, 625(7995), 548–556. https://doi.org/10.1038/s41586-023-06883-y	United States	5	7838
**10**	Badrinathan, S. (2021). Educative Interventions to Combat Misinformation: Evidence from a Field Experiment in India. American Political Science Review, 115(4), 1325–1341. https://doi.org/10.1017/S0003055421000459	India	2	406
**11**	Bago, B., Rand, D. G., & Pennycook, G. (2020). Fake news, fast and slow: Deliberation reduces belief in false (but not true) news headlines. Journal of Experimental Psychology: General, 149(8), 1608–1613. https://doi.org/10.1037/xge0000729	United States	3	561
**12**	Bago, B., Rosenzweig, L. R., Berinsky, A. J., & Rand, D. G. (2022). Emotion may predict susceptibility to fake news but emotion regulation does not seem to help. Cognition and Emotion, 1–15. https://doi.org/10.1080/02699931.2022.2090318	United States	8	4347
**13**	Basol, M., Roozenbeek, J., Berriche, M., Uenal, F., McClanahan, W. P., & Linden, S. van der. (2021). Towards psychological herd immunity: Cross-cultural evidence for two prebunking interventions against COVID-19 misinformation. Big Data & Society, 8(1), 205395172110138. https://doi.org/10.1177/20539517211013868	Europe/United States, France, Germany, UK	11	3548
**14**	Brashier, N. M., Pennycook, G., Berinsky, A. J., & Rand, D. G. (2021). Timing matters when correcting fake news. Proceedings of the National Academy of Sciences, 118(5), e2020043118. https://doi.org/10.1073/pnas.2020043118	United States	2	812
**15**	Bronstein, M. V., Pennycook, G., Bear, A., Rand, D. G., & Cannon, T. D. (2019). Belief in Fake News is Associated with Delusionality, Dogmatism, Religious Fundamentalism, and Reduced Analytic Thinking. Journal of Applied Research in Memory and Cognition, 8(1), 108–117. https://doi.org/10.1016/j.jarmac.2018.09.005	United States	2	948
**16**	Chen, X., Pennycook, G., & Rand, D. (2023). What Makes News Sharable on Social Media? Journal of Quantitative Description: Digital Media, 3. https://doi.org/10.51685/jqd.2023.007	United States	2	5000

**17**	Clayton, K., Blair, S., Busam, J. A., Forstner, S., Glance, J., Green, G., Kawata, A., Kovvuri, A., Martin, J., Morgan, E., Sandhu, M., Sang, R., Scholz-Bright, R., Welch, A. T., Wolff, A. G., Zhou, A., & Nyhan, B. (2020). Real Solutions for Fake News? Measuring the Effectiveness of General Warnings and Fact-Check Tags in Reducing Belief in False Stories on Social Media. Political Behavior, 42(4), 1073–1095. https://doi.org/10.1007/s11109-019-09533-0	United States	1	469
**18**	Clemm von Hohenberg, B. (2023). Truth and Bias, Left and Right: Testing Ideological Asymmetries with a Realistic News Supply. Public Opinion Quarterly, nfad013.	United States	1	1393
**19**	Dias, N., Pennycook, G., & Rand, D. G. (2020). Emphasizing publishers does not effectively reduce susceptibility to misinformation on social media. Harvard Kennedy School Misinformation Review. https://doi.org/10.37016/mr-2020-001	United States	3	1297
**20**	Epstein, Z., Sirlin, N., Arechar, A., Pennycook, G., & Rand, D. (2023). The social media context interferes with truth discernment. Science Advances, 9(9), eabo6169. https://doi.org/10.1126/sciadv.abo6169	United States	8	1532
**21**	Erlich, A., & Garner, C. (2023). Is pro-Kremlin Disinformation Effective? Evidence from Ukraine. The International Journal of Press/Politics, 28(1), 5–28. https://doi.org/10.1177/19401612211045221	Ukraine	8	11448
**22**	Espina Mairal, S., Bustos, F., Solovey, G., & Navajas, J. (2023). Interactive crowdsourcing to fact-check politicians. Journal of Experimental Psychology: Applied. https://doi.org/10.1037/xap0000492	Argentina	4	420
**23**	Eun-Ju Lee & Jeong-woo Jang (2023): How Political Identity and Misinformation Priming Affect Truth Judgments and Sharing Intention of Partisan News, Digital Journalism, DOI: 10.1080/21670811.2022.2163413	South Korea	8	328
**24**	Faragó, L., Krekó, P., & Orosz, G. (2023). Hungarian, lazy, and biased: The role of analytic thinking and partisanship in fake news discernment on a Hungarian representative sample. Scientific Reports, 13(1), 178. https://doi.org/10.1038/s41598-022-26724-8	Hungary	3	991
**25**	Fazio, L., Rand, D., Lewandowsky, S., Susmann, M., Berinsky, A. J., Guess, A., Kendeou, P., Lyons, B., Miller, J., Newman, E., Pennycook, G., & Swire-Thompson, B. (2024). Combating misinformation: A megastudy of nine interventions designed to reduce the sharing of and belief in false and misleading headlines. OSF. https://doi.org/10.31234/osf.io/uyjha	United States	2	1617

**26**	Garrett, R. K., & Bond, R. M. (2021). Conservatives' susceptibility to political misperceptions. Science Advances, 7(23), eabf1234. https://doi.org/10.1126/sciadv.abf1234	United States	47	1204
**27**	Gawronski, B., Ng, N. L., & Luke, D. M. (2023). Truth sensitivity and partisan bias in responses to misinformation. Journal of Experimental Psychology: General, 152(8), 2205–2236. https://doi.org/10.1037/xge0001381	United States	4	481
**28**	Gottlieb, J., Adida, C., & Moussa, R. (2022). Reducing Misinformation in a Polarized Context: Experimental Evidence from Côte d'Ivoire. OSF Preprints. https://doi.org/10.31219/osf.io/6x4wy	Ivory Coast	2	1160
**29**	Guess, A. M., Lerner, M., Lyons, B., Montgomery, J. M., Nyhan, B., Reifler, J., & Sircar, N. (2020). A digital media literacy intervention increases discernment between mainstream and false news in the United States and India. Proceedings of the National Academy of Sciences, 117(27), 15536–15545. https://doi.org/10.1073/pnas.1920498117	India	4	3508
**30**	Guess, A., McGregor, S., Pennycook, G., & Rand, D. (2024). Unbundling Digital Media Literacy Tips: Results from Two Experiments. OSF. https://doi.org/10.31234/osf.io/u34fp	United States	9	1088
**31**	Hameleers, M., Tulin, M., De Vreese, C., Aalberg, T., Van Aelst, P., Cardenal, A. S., Corbu, N., Van Erkel, P., Esser, F., Gehle, L., Halagiera, D., Hopmann, D., Koc-Michalska, K., Matthes, J., Meltzer, C., Mihelj, S., Schemer, C., Sheafer, T., Splendore, S., . . . Zoizner, A. (2023). Mistakenly misinformed or intentionally deceived? Mis- and Disinformation perceptions on the Russian War in Ukraine among citizens in 19 countries. European Journal of Political Research, 1475-6765.12646. https://doi.org/10.1111/1475-6765.12646	Austria, Belgium, Brazil, Czech Republic, Denmark, France, Germany, Great Britain, Greece, Hungary, Italy, Netherlands, Poland, Romania, Serbia, Spain, Sweden, Switzerland, United States	19	19037
**32**	Hlatky, R. (2024). Unintended Consequences? Russian Disinformation and Public Opinion. OSF. https://doi.org/10.31219/osf.io/85vmt	Slovakia	2	961
**33**	Kirill Bryanov, Reinhold Kliegl, Olessia Koltsova, Tetyana Lokot, Alex Miltsov, Sergei Pashakhin, Alexander Porshnev, Yadviga Sinyavskaya, Maksim Terpilovskii & Victoria Vziatysheva (2023) What Drives Perceptions of Foreign News Coverage Credibility? A Cross- National Experiment Including Kazakhstan, Russia, and Ukraine, Political Communication, 40:2, 115-146, DOI: 10.1080/10584609.2023.2172492	Kazakhstan, Russia, Ukraine	5	8559

**34**	Koetke, J., Schumann, K., Porter, T., & Smilo-Morgan, I. (2023). Fallibility Salience Increases Intellectual Humility: Implications for People's Willingness to Investigate Political Misinformation. Personality and Social Psychology Bulletin, 49(5), 806–820. https://doi.org/10.1177/01461672221080979	United States	1	289
**35**	Kreps, S. E., & Kriner, D. L. (2023). Assessing misinformation recall and accuracy perceptions: Evidence from the COVID-19 pandemic. Harvard Kennedy School Misinformation Review. https://doi.org/10.37016/mr-2020-123	United States	1	1045
**36**	Luo, M., Hancock, J. T., & Markowitz, D. M. (2022). Credibility Perceptions and Detection Accuracy of Fake News Headlines on Social Media: Effects of Truth-Bias and Endorsement Cues. Communication Research, 49(2), 171–195. https://doi.org/10.1177/0093650220921321	United States	3	337
**37**	Lutzke, L., Drummond, C., Slovic, P., & Árvai, J. (2019). Priming critical thinking: Simple interventions limit the influence of fake news about climate change on Facebook. Global Environmental Change, 58, 101964. https://doi.org/10.1016/j.gloenvcha.2019.101964	United States	1	934
**38**	Lyons, B., King, A. J., & Kaphingst, K. (2024). A health media literacy intervention increases skepticism of both inaccurate and accurate cancer news among U.S. adults. https://doi.org/10.31219/osf.io/hm9ty	United States	1	195
**39**	Lyons, B., Modirrousta-Galian, A., Altay, S., & Salovich, N. A. (2024). Reduce blind spots to improve news discernment? Performance feedback reduces overconfidence but does not improve subsequent discernment. https://doi.org/10.31219/osf.io/kgfrb	United States	12	2194
**40**	Lyons, B., Montgomery, J., & Reifler, J. (2023). Partisanship and older Americans' engagement with dubious political news. OSF. https://doi.org/10.31219/osf.io/etb89	United States	2	2454
**41**	Lühring, J., Shetty, A., Koschmieder, C., Garcia, D., Waldherr, A., & Metzler, H. (2023). Emotions in misinformation studies: Distinguishing affective state from emotional response and misinformation recognition from acceptance. PsyArXiv. https://doi.org/10.31234/osf.io/udqms	Austria	1	422
**42**	Maertens, R., Götz, F. M., Schneider, C. R., Roozenbeek, J., Kerr, J. R., Stieger, S., McClanahan, W. P., Drabot, K., & Linden, S. van der. (2021). The Misinformation Susceptibility Test (MIST): A psychometrically validated measure of news veracity discernment [Preprint]. PsyArXiv. https://doi.org/10.31234/osf.io/gk68h	UK, United States	2	6461

**43**	Martel, C., Pennycook, G., & Rand, D. G. (2020). Reliance on emotion promotes belief in fake news. Cognitive Research: Principles and Implications, 5(1), 47. https://doi.org/10.1186/s41235-020-00252-3	United States	2	1704
**44**	Modirrousta-Galian, A., Higham, P. A., & Seabrooke, T. (2023). Effects of inductive learning and gamification on news veracity discernment. Journal of Experimental Psychology: Applied, 29(3), 599–619. https://doi.org/10.1037/xap0000458	United States	1	72
**45**	Modirrousta-Galian, A., Higham, P. A., & Seabrooke, T. (2024). Wordless wisdom: The dominant role of tacit knowledge in true and fake news discrimination. Journal of Applied Research in Memory and Cognition.	United States	1	327
**46**	Muda, R., Pennycook, G., Hamerski, D., & Białek, M. (2023). People are worse at detecting fake news in their foreign language. Journal of Experimental Psychology: Applied, 29(4), 712–724. https://doi.org/10.1037/xap0000475	Poland	2	318
**47**	OECD. (2022). An international effort using behavioural science to tackle the spread of misinformation (OECD Public Governance Policy Papers 21; OECD Public Governance Policy Papers, Vol. 21). https://doi.org/10.1787/b7709d4f-en	Canada	1	282
**48**	Orosz, G., Paskuj, B., Faragó, L., & Krekó, P. (2023). A prosocial fake news intervention with durable effects. Scientific Reports, 13(1), 3958. https://doi.org/10.1038/s41598-023-30867-7	Hungary	2	412
**49**	Pehlivanoglu, D., Lin, T., Deceus, F., Heemskerk, A., Ebner, N. C., & Cahill, B. S. (2021). The role of analytical reasoning and source credibility on the evaluation of real and fake full-length news articles. Cognitive Research: Principles and Implications, 6(1), 24. https://doi.org/10.1186/s41235-021-00292-3	United States	4	649
**50**	Pennycook, G., & Rand, D. G. (2019). Lazy, not biased: Susceptibility to partisan fake news is better explained by lack of reasoning than by motivated reasoning. Cognition, 188, 39–50. https://doi.org/10.1016/j.cognition.2018.06.011	United States	1	2644
**51**	Pennycook, G., & Rand, D. G. (2020). Who falls for fake news? The roles of bullshit receptivity, overclaiming, familiarity, and analytic thinking. Journal of Personality, 88(2), 185–200. https://doi.org/10.1111/jopy.12476	United States	2	1204
**52**	Pennycook, G., Binnendyk, J., Newton, C., & Rand, D. G. (2021). A Practical Guide to Doing Behavioral Research on Fake News and Misinformation. Collabra: Psychology, 7(1), 25293. https://doi.org/10.1525/collabra.25293	United States	2	2013

**53**	Pennycook, G., Cannon, T. D., & Rand, D. G. (2018). Prior exposure increases perceived accuracy of fake news. Journal of Experimental Psychology: General, 147(12), 1865–1880. https://doi.org/10.1037/xge0000465	United States	2	474
**54**	Pennycook, G., McPhetres, J., Zhang, Y., Lu, J. G., & Rand, D. G. (2020). Fighting COVID-19 Misinformation on Social Media: Experimental Evidence for a Scalable Accuracy-Nudge Intervention. 11.	United States	1	426
**55**	Pereira, F. B., Bueno, N. S., Nunes, F., & Pavão, N. (2023). Inoculation Reduces Misinformation: Experimental Evidence from Multidimensional Interventions in Brazil. Journal of Experimental Political Science, 1–12. https://doi.org/10.1017/XPS.2023.11	Brazil	3	1037
**56**	Peren Arin, K., Mazrekaj, D., & Thum, M. (2023). Ability of detecting and willingness to share fake news. Scientific Reports, 13(1), 7298.	Germany, UK	2	2379
**57**	Rathje, S., Roozenbeek, J., Van Bavel, J.J. et al. Accuracy and social motivations shape judgements of (mis)information. Nat Hum Behav (2023). https://doi.org/10.1038/s41562-023-01540-w	United States	10	1303
**58**	Roozenbeek, J., Maertens, R., Herzog, S. M., Geers, M., Kurvers, R., & Sultan, M. (2022). Susceptibility to misinformation is consistent across question framings and response modes and better explained by myside bias and partisanship than analytical thinking. Judgment and Decision Making, 17(3), 27.	United States	8	2622
**59**	Roozenbeek, J., Schneider, C. R., Dryhurst, S., Kerr, J., Freeman, A. L. J., Recchia, G., van der Bles, A. M., & van der Linden, S. (2020). Susceptibility to misinformation about COVID-19 around the world. Royal Society Open Science, 7(10), 201199. https://doi.org/10.1098/rsos.201199	Ireland, Mexico, Spain, UK, United States	6	5000
**60**	Rosenzweig, L. R., Bago, B., Berinsky, A. J., & Rand, D. G. (2021). Happiness and surprise are associated with worse truth discernment of COVID-19 headlines among social media users in Nigeria. Harvard Kennedy School Misinformation Review. https://doi.org/10.37016/mr-2020-75	Nigeria	1	1341
**61**	Ross, B., Heisel, J., Jung, A.-K., & Stieglitz, S. (2018). Fake News on Social Media: The (In)Effectiveness of Warning Messages.	Germany	1	53
**62**	Ross, R. M., Rand, D. G., & Pennycook, G. (2021). Beyond “fake news”: Analytic thinking and the detection of false and hyperpartisan news headlines. Judgment and Decision Making, 16(2), 22.	United States	2	940

**63**	Shirikov, A. (2024). Fake News for All: How Citizens Discern Disinformation in Autocracies. Political Communication, 41(1), 45–65. https://doi.org/10.1080/10584609.2023.2257618	Russia	8	51764
**64**	Smelter, T. J., & Calvillo, D. P. (2020). Pictures and repeated exposure increase perceived accuracy of news headlines. Applied Cognitive Psychology, 34(5), 1061–1071. https://doi.org/10.1002/acp.3684	United States	5	594
**65**	Stagnaro, M., Pink, S., Rand, D. G., & Willer, R. (2023). Increasing accuracy motivations using moral reframing does not reduce Republicans' belief in false news. Harvard Kennedy School Misinformation Review. https://doi.org/10.37016/mr-2020-128	United States	1	1007
**66**	Sultan, M., Tump, A. N., Geers, M., Lorenz-Spreen, P., Herzog, S. M., & Kurvers, R. H. J. M. (2022). Time pressure reduces misinformation discrimination ability but does not alter response bias. Scientific Reports, 12(1), 22416. https://doi.org/10.1038/s41598-022-26209-8	United States	2	382
**67**	Winter, S., Valenzuela, S., Santos, M., Schreyer, T., Iwertowski, L., & Rothmund, T. (2024). (Don't) Stop Believing: A Signal Detection Approach to Risk and Protective Factors for Engagement with Politicized (Mis)Information in Social Media.	Germany	1	992
