# Supplementary material for: Spotting false news and doubting true news: a systematic review and meta-analysis of news judgements
Source: Nat Hum Behav. 2025 Feb 21;9(4):688–99. doi: 10.1038/s41562-024-02086-1 (PMC12018262; doi:10.1038/s41562-024-02086-1)
Supplement: Supplementary file 2 — Reporting Summary [file 41562_2024_2086_MOESM2_ESM.pdf]

nature portfolio

Corresponding author(s): Sacha Altay
Last updated by author(s): Oct 21, 2024

Reporting Summary

Nature Portfolio wishes to improve the reproducibility of the work that we publish. This form provides structure for consistency and transparency in reporting. For further information on Nature Portfolio policies, see our Editorial Policies and the Editorial Policy Checklist.

Statistics

For all statistical analyses, confirm that the following items are present in the figure legend, table legend, main text, or Methods section.

n/a	Confirmed	
☐	☒	The exact sample size (*n*) for each experimental group/condition, given as a discrete number and unit of measurement
☐	☒	A statement on whether measurements were taken from distinct samples or whether the same sample was measured repeatedly
☐	☒	The statistical test(s) used AND whether they are one- or two-sided<br>*Only common tests should be described solely by name; describe more complex techniques in the Methods section.*
☐	☒	A description of all covariates tested
☐	☒	A description of any assumptions or corrections, such as tests of normality and adjustment for multiple comparisons
☐	☒	A full description of the statistical parameters including central tendency (e.g. means) or other basic estimates (e.g. regression coefficient) AND variation (e.g. standard deviation) or associated estimates of uncertainty (e.g. confidence intervals)
☐	☒	For null hypothesis testing, the test statistic (e.g. *F*, *t*, *r*) with confidence intervals, effect sizes, degrees of freedom and *P* value noted<br>*Give P values as exact values whenever suitable.*
☒	☐	For Bayesian analysis, information on the choice of priors and Markov chain Monte Carlo settings
☐	☒	For hierarchical and complex designs, identification of the appropriate level for tests and full reporting of outcomes
☐	☒	Estimates of effect sizes (e.g. Cohen's *d*, Pearson's *r*), indicating how they were calculated

Our web collection on statistics for biologists contains articles on many of the points above.

Software and code

Policy information about availability of computer code

Data collection	We used R version 4.2.2 (2022-10-31), Rstudio version 2024.9.0.375, and the tidyverse package version 2.0.068 for extracting data from papers.The extracted data used to produce our results are available on the OSF project page (https://osf.io/96zbp/).
Data analysis	We conducted all analyses in R version 4.2.2 (2022-10-31) using Rstudio version 2024.9.0.375 and the tidyverse package version 2.0.068. For effect size calculations, we rely on the escalc(), for models on therma.mv(), for clustered standard errors on the robust() function, all from the metafor package version 4.6.069. The code used to create all results (including tables and figures) of this manuscript is also available on the OSF project page (https://osf.io/96zbp/).

For manuscripts utilizing custom algorithms or software that are central to the research but not yet described in published literature, software must be made available to editors and reviewers. We strongly encourage code deposition in a community repository (e.g. GitHub). See the Nature Portfolio guidelines for submitting code & software for further information.

Data

Policy information about availability of data

All manuscripts must include a data availability statement. This statement should provide the following information, where applicable:

- Accession codes, unique identifiers, or web links for publicly available datasets
- A description of any restrictions on data availability
- For clinical datasets or third party data, please ensure that the statement adheres to our policy

The extracted data used to produce our results are available on the OSF project page (https://osf.io/96zbp/). For our literature search, we relied on the Scopus database (https://www.scopus.com/search/form.uri?display=basic#basic).

Research involving human participants, their data, or biological material

Policy information about studies with human participants or human data. See also policy information about sex, gender (identity/presentation), and sexual orientation and race, ethnicity and racism.

Reporting on sex and gender	No reporting on sex or gender.
Reporting on race, ethnicity, or other socially relevant groupings	No reporting on race, ethnicity, or other socially relevant groupings.
Population characteristics	No population covariates were measured, except for country.
Recruitment	Most studies included in our meta-analysis recruited participants online, some of which used representative samples.
Ethics oversight	None.

Note that full information on the approval of the study protocol must also be provided in the manuscript.

Field-specific reporting

Please select the one below that is the best fit for your research. If you are not sure, read the appropriate sections before making your selection.

☐ Life sciences ☒ Behavioural & social sciences ☐ Ecological, evolutionary & environmental sciences

For a reference copy of the document with all sections, see nature.com/documents/nr-reporting-summary-flat.pdf

Behavioural & social sciences study design

All studies must disclose on these points even when the disclosure is negative.

Study description	We conducted a meta-analysis, i.e. took a purely quantitative approach in our systematic review.
Research sample	We selected all our studies systematically, following the PRISMA guidelines. Most samples of included studies were recruited online, via platforms such as Amazon MTurk or Prolific. Some studies recruited representative samples of the respective country population.
Sampling strategy	We selected all our studies systematically, following the PRISMA guidelines. Our inclusion criteria are documented in the article.
Data collection	We retrieved the relevant summary statistics directly from the paper for 21 papers, calculated them ourselves based on publicly available raw data for 31 papers, and got them from the authors after request for 15 papers. For the second systematic search, both authors screened search results independently. In case of conflicting decisions, an article passed onto the next stage (i.e. receive abstract screening or full text assessment).
Timing	We conducted our first search on March 2, 2023. After submitting a manuscript version, reviewers remarked that not including the terms “misinformation” or “disinformation” in our search string might have omitted relevant results. On March 22nd, 2024, we therefor conducted a second, pre-registered (https://osf.io/yn6r2) search using an extended query string.
Data exclusions	We document all exclusion phases following the PRISMA protocol in our article. Specifically, we stored 264 records in the reference management system Zotero for retrieval. Of those, we rejected a total of 217 papers that did not meet our inclusion criteria. We rejected 87 papers based on their abstract and 130 after assessment of the full text. All exclusion decisions are documented in a publicly available spreadsheet that can be found on the OSF project page (https://osf.io/96zbp/).
Non-participation	We rely on published data and that information was not available in most cases.
Randomization	Almost all studies used a within-participant design. Between-participant studies randomized participants to see either true or false

Randomization | news.

Reporting for specific materials, systems and methods

We require information from authors about some types of materials, experimental systems and methods used in many studies. Here, indicate whether each material, system or method listed is relevant to your study. If you are not sure if a list item applies to your research, read the appropriate section before selecting a response.

Materials & experimental systems

n/a	Involved in the study
☒	☐ Antibodies
☒	☐ Eukaryotic cell lines
☒	☐ Palaeontology and archaeology
☒	☐ Animals and other organisms
☒	☐ Clinical data
☒	☐ Dual use research of concern
☒	☐ Plants

Methods

n/a	Involved in the study
☒	☐ ChIP-seq
☒	☐ Flow cytometry
☒	☐ MRI-based neuroimaging

Plants

Seed stocks

Report on the source of all seed stocks or other plant material used. If applicable, state the seed stock centre and catalogue number. If plant specimens were collected from the field, describe the collection location, date and sampling procedures.

Novel plant genotypes

Describe the methods by which all novel plant genotypes were produced. This includes those generated by transgenic approaches, gene editing, chemical/radiation-based mutagenesis and hybridization. For transgenic lines, describe the transformation method, the number of independent lines analyzed and the generation upon which experiments were performed. For gene-edited lines, describe the editor used, the endogenous sequence targeted for editing, the targeting guide RNA sequence (if applicable) and how the editor was applied.

Authentication

Describe any authentication procedures for each seed stock used or novel genotype generated. Describe any experiments used to assess the effect of a mutation and, where applicable, how potential secondary effects (e.g. second site T-DNA insertions, mosiacism, off-target gene editing) were examined.
